# Brain scaling in mammalian evolution as a consequence of concerted and mosaic changes in numbers of neurons and average neuronal cell size

**DOI:** 10.3389/fnana.2014.00077

**Published:** 2014-08-11

**Authors:** Suzana Herculano-Houzel, Paul R. Manger, Jon H. Kaas

**Affiliations:** ^1^Instituto de Ciências Biomédicas, Universidade Federal do Rio de JaneiroRio de Janeiro, Brazil; ^2^Instituto Nacional de Neurociência Translacional, Ministério de Ciência e TecnologiaSão Paulo, Brazil; ^3^Department of Anatomy, University of the WitwatersrandJohannesburg, South Africa; ^4^Department of Psychology, Vanderbilt UniversityNashville, TN, USA

**Keywords:** numbers of neurons, brain size, cortical expansion, evolution, cell size

## Abstract

Enough species have now been subject to systematic quantitative analysis of the relationship between the morphology and cellular composition of their brain that patterns begin to emerge and shed light on the evolutionary path that led to mammalian brain diversity. Based on an analysis of the shared and clade-specific characteristics of 41 modern mammalian species in 6 clades, and in light of the phylogenetic relationships among them, here we propose that ancestral mammal brains were composed and scaled in their cellular composition like modern afrotherian and glire brains: with an addition of neurons that is accompanied by a decrease in neuronal density and very little modification in glial cell density, implying a significant increase in average neuronal cell size in larger brains, and the allocation of approximately 2 neurons in the cerebral cortex and 8 neurons in the cerebellum for every neuron allocated to the rest of brain. We also propose that in some clades the scaling of different brain structures has diverged away from the common ancestral layout through clade-specific (or clade-defining) changes in how average neuronal cell mass relates to numbers of neurons in each structure, and how numbers of neurons are differentially allocated to each structure relative to the number of neurons in the rest of brain. Thus, the evolutionary expansion of mammalian brains has involved both concerted and mosaic patterns of scaling across structures. This is, to our knowledge, the first mechanistic model that explains the generation of brains large and small in mammalian evolution, and it opens up new horizons for seeking the cellular pathways and genes involved in brain evolution.

## Introduction

In contrast to ancestral mammalian brains, which were small and lissencephalic (Luo et al., 2001; Rowe et al., 2011), modern mammalian brains vary over 100,000-fold in mass (Count, 1947), although not uniformly: members of different clades can be distinguished by the relative volume of brain structures as well as by other morphological aspects, such as the layout and extent of cortical folds (Welker, 1990; Pillay and Manger, 2007). What events in evolution have led to the different patterns of brain expansion across mammalian clades?

Mammalian brain evolution has often been regarded as a simple issue of brain expansion, or rather cerebral cortical expansion, the most obvious feature that accompanies this evolution (Hofman, 1985; Finlay and Darlington, 1995; Rakic, 1995; Rowe et al., 2011). One basic assumption in previous studies of brain scaling in evolution was that the same rules applied equally to all mammalian species: as brains increased in volume (or rather, the cerebral cortex, on which most studies focused), the cerebral cortex supposedly expands homogeneously in surface area across species (Hofman, 1985—even if different functional areas expand heterogeneously within that surface, e.g., Chaplin et al., 2013), with decreasing neuronal densities and an increasing glia/neuron ratio (Tower and Elliott, 1952; Tower, 1954; Hawkins and Olszewski, 1957; Prothero and Sundsten, 1984; Haug, 1987). Based on the same assumption, other studies (Finlay and Darlington, 1995; Rakic, 1995; Lui et al., 2011; Charvet et al., 2013) have concentrated on explaining how increased numbers of neurons in the developing cerebral cortex would result in cortical expansion across rodents, artiodactyls and primates alike.

It is clear from comparative volumetric studies, however, that expansion of the cerebral cortex as a whole has not been homogeneous with expanding brain volume, given that mammalian brains of a similar total volume can have different proportions allocated to the cerebral cortex (Frahm et al., 1982; Clark et al., 2001). Additionally, our recent studies have shown that cortical expansion is not a simple function of the addition of neurons to the cerebral cortex, as artiodactyl and primate cortices of similar cortical surface areas have remarkably different numbers of neurons (Kazu et al., under review), and cortical folding scales differently with numbers of cortical neurons across afrotherians, glires, primates and artiodactyls (Herculano-Houzel et al., 2010; Ventura-Antunes et al., 2013; Neves et al., 2014; Kazu et al., under review). Moreover, studies on brain evolution have so far failed to offer mechanistic accounts of how brain morphology could vary as brains become larger in evolution, for instance with different degrees of relative cortical expansion and different relative cerebellar volumes across clades (Frahm et al., 1982; Clark et al., 2001; Maseko et al., 2012). Such clade-specific aspects of brain morphology have been neglected by studies focused on linked regularities across brain structure volumes in brain scaling (Finlay and Darlington, 1995), while it is evident that clade-specific patterns exist in the volume relationships across brain structures (Frahm et al., 1982; Barton and Harvey, 2000; Clark et al., 2001), which has been referred to as mosaic evolution.

Another problem in the comparative evolutionary neuroscience literature has been the common use of brain size (volume or mass) as an independent variable against which other parameters are compared across species (e.g., Tower and Young, 1973; Prothero and Sundsten, 1984; Hofman, 1985; Haug, 1987; Finlay and Darlington, 1995; Karbowski, 2007). While the use of brain size as an independent variable has useful descriptive power, it implicitly or sometimes explicitly assumes that total brain volume actually determines changes in neuronal density and even the size of various brain parts. This is obviously not the case, as total adult brain size can only be a consequence of the sizes of its component structures. In the body of work reviewed here, we have explicitly considered brain mass (or brain structure mass) as a dependent variable, and the same applies to the model we propose, which explains variations in brain size (mass) as consequences of evolutionary changes in numbers of cells and average cell size.

In this review, we examine the variation in several aspects of the cellular composition of mammalian brains and propose a small suite of mechanisms that suffice to explain the evolutionary generation of diversity in brain size and morphology across clades. The basic underlying concept in this review is that brain size is a joint consequence of the numbers of cells that build a structure and the average size of those cells: if numbers of cells and/or their average size change (and by size we mean the dimensions of the entire cell, including soma and all arborizations), then brain structure size changes as a result. We will first analyze how numbers of neuronal and non-neuronal cells and their densities, which we consider as the primary parameters that are subject to change in evolution, vary and scale across mammalian species. Changes in the proportions of neurons across structures will be considered second, and only then we will address evolutionary changes in brain mass and in the proportions of brain structures as consequences of changes in the cellular composition of brain structures and in the relative distribution of neurons across them.

Contrary to the assumption that all brains scale the same way, with homogeneous, regular changes in the relationship between structure mass and number of neurons and neuronal density, our studies using the isotropic fractionator to quantify cell populations in brain structures across mammalian species showed that there is much variation across clades (reviewed in Herculano-Houzel, 2011). Remarkably, however, the relationship between numbers of neurons and the size (mass) of brain structures (which we refer to as the neuronal scaling rule for each structure) is not entirely clade-specific: while the closely related glires and primates exhibit markedly different neuronal scaling rules (Herculano-Houzel et al., 2006, 2007, 2011; Gabi et al., 2010), the more evolutionarily distant afrotherians and artiodactyls share scaling rules not only amongst themselves but also with glires (Neves et al., 2014; Kazu et al., under review).

Here we analyze the shared and clade-specific neuronal scaling rules that apply to each of 6 mammalian clades in the light of the branching patterns in mammalian evolution. We look for commonalities across modern species in the different clades to infer the scaling rules that applied to mammalian ancestors prior to the divergence of each clade, and we look for clade-specific characteristics as clues to the events that led to the separation of each lineage in evolution. We use exclusively the dataset generated in collaboration by our labs using the isotropic fractionator (Herculano-Houzel and Lent, 2005), which is the only dataset that analyzes simultaneously the cellular composition of different parts of the brain (as opposed to only the cerebral cortex, the structure for which most independently generated data are available). The isotropic fractionator has been shown to generate results similar to those obtained with unbiased stereology (Bahney and von Bartheld, 2014; Miller et al., 2014).

**Figure 1:**
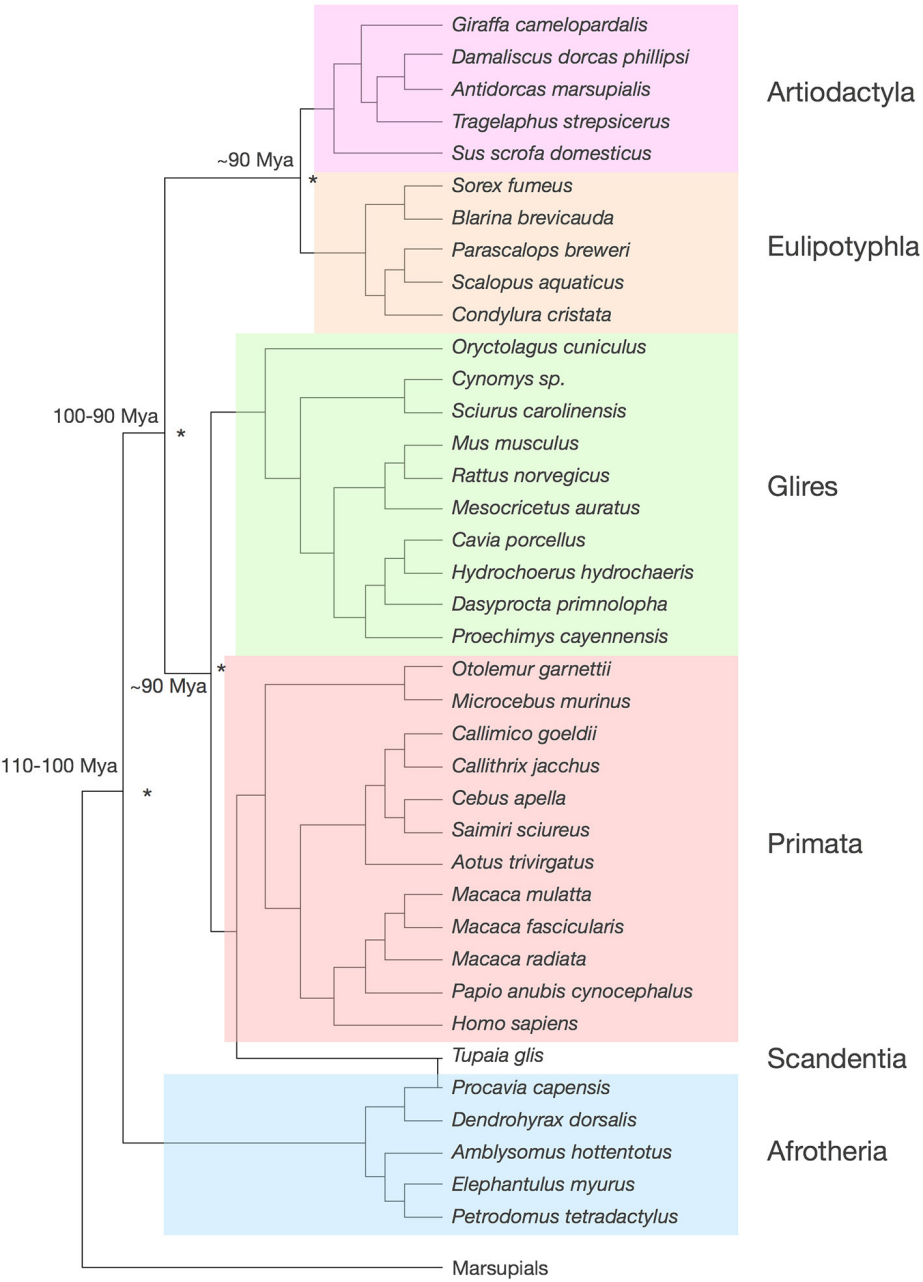
**Phylogenetic relationships among the 41 species analyzed**. Tree compiled according to Price et al. (2005); Purvis (1995); Blanga-Kanfi et al. (2009); Douady et al. (2002); Shinohara et al. (2003). The same color code identifying clades is used throughout the figures. Estimated times of divergence across clades (asterisks) are indicated, according to Murphy et al. (2004).

Our dataset includes the cerebral cortex (both gray and white matter combined), the cerebellum (including the deep nuclei), the olfactory bulb and the rest of brain (the ensemble of brainstem, diencephalon and striatum). We analyze a total of 41 species, whose phylogenetic relationships are shown in Figure 1. Our data include 5 afrotherians (Neves et al., 2014), 10 glires (Herculano-Houzel et al., 2006, 2011), 15 primates (including humans; Herculano-Houzel et al., 2007; Azevedo et al., 2009; Gabi et al., 2010), 5 eulipotyphlans (Sarko et al., 2009), 1 scandentia (Herculano-Houzel et al., 2007) and 5 artiodactyls (Kazu et al., under review). Afrotherians are an important group for the phylogenetic analysis of brain scaling due to their basal branching between 100 and 110 million years ago from the lineages that gave rise to laurasiatherians (artiodactyls and eulipotyphlans, in our sample) and to euarchontoglires (rodents, lagomorphs, scandentia and primates; Murphy et al., 2004). Laurasiatherians and euarchontoglires diverged 90–100 million years ago, and within each of them, both artiodactyls and eulipotyphlans, and glires and primates, branched off around 90 million years ago (Murphy et al.; Figure 1). Thus, although our analysis at present excludes carnivores, perissodactyls, chiropterans, and cetaceans (although the latter belong to the order Cetartiodactyla, which includes the artiodactyls examined here), all these clades are part of Laurasiatheria; Xenarthra is the only major independent branch of eutherians that is missing in our analysis.

## Estimating changes in average neuronal cell size

While the isotropic fractionator provides reliable estimates of total numbers of neuronal and non-neuronal cells in different brain structures, it does not measure cell size. However, it does measure neuronal and non-neuronal cell densities, and the inverse of neuronal density can be used as a proxy for average neuronal cell size (which includes the cell body, the entire axon, and all dendritic arbors). This is because non-neuronal density varies little in comparison to variations in neuronal density, both across structures and across species. The small variation in non-neuronal cell density across structures and species, as will be reviewed below, implies that average glial cell size varies only modestly, and non-systematically, across brain structures and species (Herculano-Houzel, 2011, 2014).

Because non-neuronal cell density varies little, neuronal density can be approximated as the inverse of average neuronal size: the larger the average size of neurons (cell bodies and arbors), the fewer the neurons that will be found per volume, and therefore the smaller the neuronal density. The situation is akin to a bowl containing peaches of different sizes, each with a central pit. The number of peaches divided by the volume of the bowl is the density of peaches in the bowl, which can be measured by counting the pits in the bowl (much like we determine the number of cell nuclei per volume or mass of brain tissue). If all peaches become much larger, the density of peaches in the bowl will necessarily become smaller. This will happen regardless of how exactly peaches become larger: if only the pit becomes larger, if peaches increase in volume while changing shape or do so isometrically, or any combination thereof, and also regardless of whether all peaches or only a particular type become larger. The density of peaches in the bowl will always necessarily be proportional to the inverse of the average size of all peaches. Thus, the ratio between the number of pits seen in the bowl and the volume of the bowl serves as a proxy for the inverse of the average size of the peaches as a whole—and it is a similar mathematical necessity that neuronal density in brain tissue serves as a proxy for the inverse of average neuronal size.

Notice, however, that measured neuronal cell density does not inform on how neurons grow—if by increasing cell body volume, arbor volume, or both. Thus, using neuronal densities to infer changes in average neuronal cell size does only that: inform how the average total volume of neuronal cells (cell body and all arbors) varies across brain structures and species. It allows no inferences about how dendritic arbors, axonal arbors and cell bodies vary or scale across species, nor does it inform about variations within a single structure, such as the cerebral cortex; that type of information requires the direct measurement and comparison of arbor size, as done independently by several groups and in several species (Jacobs et al., 2001, 2014; Elston, 2003; Benavides-Piccione et al., 2006; Bianchi et al., 2012; Oga et al., 2013; Elston and Manger, 2014).

A recent mathematical model that employed chi-square minimization of variations in neuronal and non-neuronal cell densities across brain structures and species to estimate average cell size found that the average mass of individual neuronal cells in a brain structure can be determined for any species and brain structure as 0.649 × neuronal density^−1.004^ (Mota and Herculano-Houzel, under review). Thus, average neuronal cell mass is proportional to the inverse of the measured neuronal cell density in brain structures, and variations in neuronal density across brain structures and species can be used as a valid proxy for variations in average neuronal size (including soma, dendritic and axonal arbors). Finally, we consider that variations in neuronal cell density, the parameter measured, are a consequence of variations in average neuronal cell size in the opposite direction, that is, that the primary variable of evolutionary change is average neuronal cell size, not neuronal density.

The analysis presented here allows us to identify what may have been the ancestral scaling rules that applied to early mammals and still apply to some modern mammalian clades. Further, we propose that the scaling of different brain structures in some clades (primates, artiodactyls, and eulipotyphlans) has diverged away from the common ancestral layout through clade-specific (or clade-defining) changes in how average neuronal cell mass relates to numbers of neurons in each structure, and how numbers of neurons are differentially allocated to each structure relative to the number of neurons in the ensemble of structures from brainstem to striatum.

## What stays the same in mammalian brain evolution: non-neuronal scaling rules

**Figure 2:**
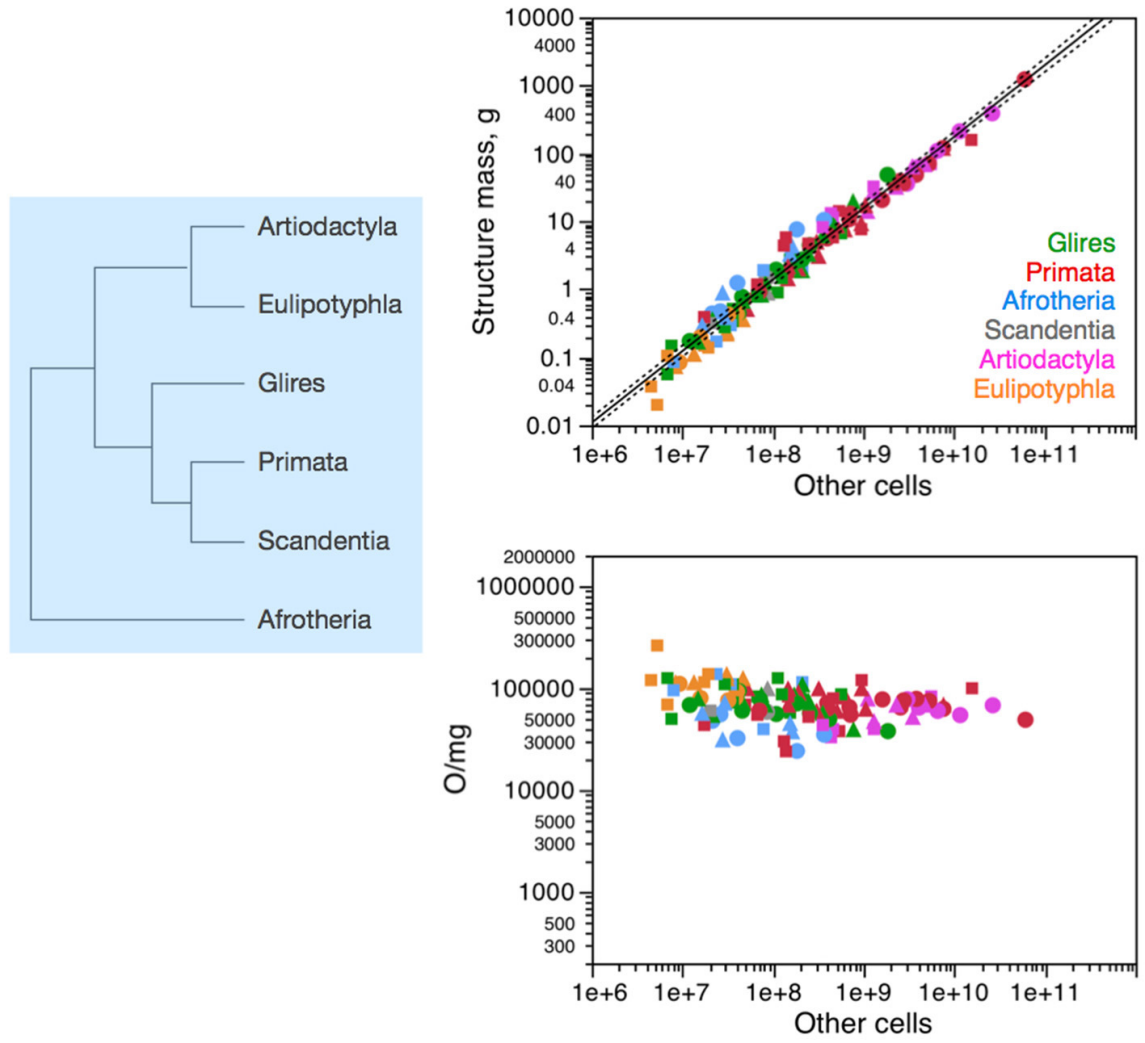
**Non-neuronal scaling rules for the different brain structures, that is, the relationship between structure mass and number of non-neuronal (other) cells, is shared across the 41 species in 6 mammalian clades, and thus presumably applied in the evolutionary history of these clades since their common ancestor**. **Top right:** scaling of brain structure mass as a function of numbers of non-neuronal (other) cells in the structure, with a common exponent of 1.020 ± 0.026, *p* < 0.0001, plotted along with the 95% confidence interval (dashed lines). **Bottom right:** variation in other (non-neuronal) cell density plotted as a function of numbers of other cells in the structure, showing no significant correlation across the parameters. Each symbol represents the average values for one brain structure (cerebral cortex, circles; cerebellum, squares; rest of brain, triangles) in one species (afrotherians, blue; glires, green; eulipotyphlans, orange; primates, red; scandentia, gray; artiodactyls, pink). The phylogenetic scheme on the left indicates the clades that share the same non-neuronal scaling rules, and the presumed extension of these shared scaling rules to the common ancestor to the 6 clades.

Across all 41 species of afrotherians, glires, eulipotyphlans, scandentia, primates and artiodactyls, the mass of each brain structure is found to vary as a similar, shared power function of the number of non-neuronal (other) cells in the structure of exponent 1.020 ± 0.026, *p* < 0.0001; Figure 2, top right). Non-neuronal cells are thus added to all brain structures in a fashion that is shared across structures (Figure 2, left). The near-linearity of the scaling of brain structure mass with numbers of non-neuronal cells is due to very small variations in non-neuronal cell density, which are also non-systematic across structures and species (Figure 2, bottom right). We have proposed that the mechanism that leads to the similar scaling of brain structure mass with numbers of non-neuronal cells is the matching of numbers of non-neuronal cells, whose average mass varies little, to the total neuronal mass in the developing tissue (Herculano-Houzel, 2011, 2014; Mota and Herculano-Houzel, under review). The evolutionary implication of the shared non-neuronal scaling rules across the clades examined here is that the mechanism that regulates the addition of glial and endothelial cells to brain structures has been conserved in evolution, and therefore the rules that apply to modern clades and are shared by them can be inferred to have also been the rules that applied to ancestral eutherian mammals over 110 million years ago (Figure 2, left), and possibly already to the last common ancestor that gave rise to mammals, about 230 million years ago (Murphy et al., 2001, 2004).

## What changes in mammalian brain evolution: neuronal scaling rules

Previously, it was implicitly assumed that all brain structures scaled in the same manner across all mammalian species, with a shared relationship between brain structure mass and numbers of neurons across all species, similarly to what we found to apply regarding non-neuronal cells (reviewed in Herculano-Houzel, 2011). Initially, upon finding that different neuronal scaling rules applied to the closely related rodents and primates (Herculano-Houzel et al., 2006; Herculano-Houzel et al., 2007), and later to eulipotyphlans (Sarko et al., 2009), we expected each mammalian order to have its set of characteristic, distinguishing neuronal scaling rules. However, the recent extension of the analysis to more distant groups—Afrotheria (Neves et al., 2014) and Artiodactyla (Kazu et al., under review), at the base and top branches of the eutherian evolutionary tree, respectively—showed that the relationship between brain structure mass and its number of composing neurons is actually shared in several ways across some mammalian clades, while being indeed distinctive in others. When viewed in the light of evolution, the patterns of shared and distinct characteristics point respectively to what were presumably the scaling rules that applied to early eutherians, and which still apply to some mammalian clades; and to watershed events in mammalian evolution that led to brains with distinctive characteristics in some mammalian clades.

The cerebral cortex of the mammalian species examined so far, which varies in mass 12,330-fold between the smoky shrew (0.1 g) and humans (1233 g), is composed of numbers of neurons that vary only 1633-fold, between 9.8 million (in the smoky shrew, a eulipotyphlan) and 16 billion in the human cortex. The cerebellum has many more neurons, varying 3285-fold between 21 million in the smoky shrew and an average of 69 billion neurons in humans; while the rest of brain (brainstem to basal ganglia), in contrast, has fewer neurons, varying only 115-fold between 6 million neurons in the smoky shrew and 690 million neurons in humans.

**Figure 3:**
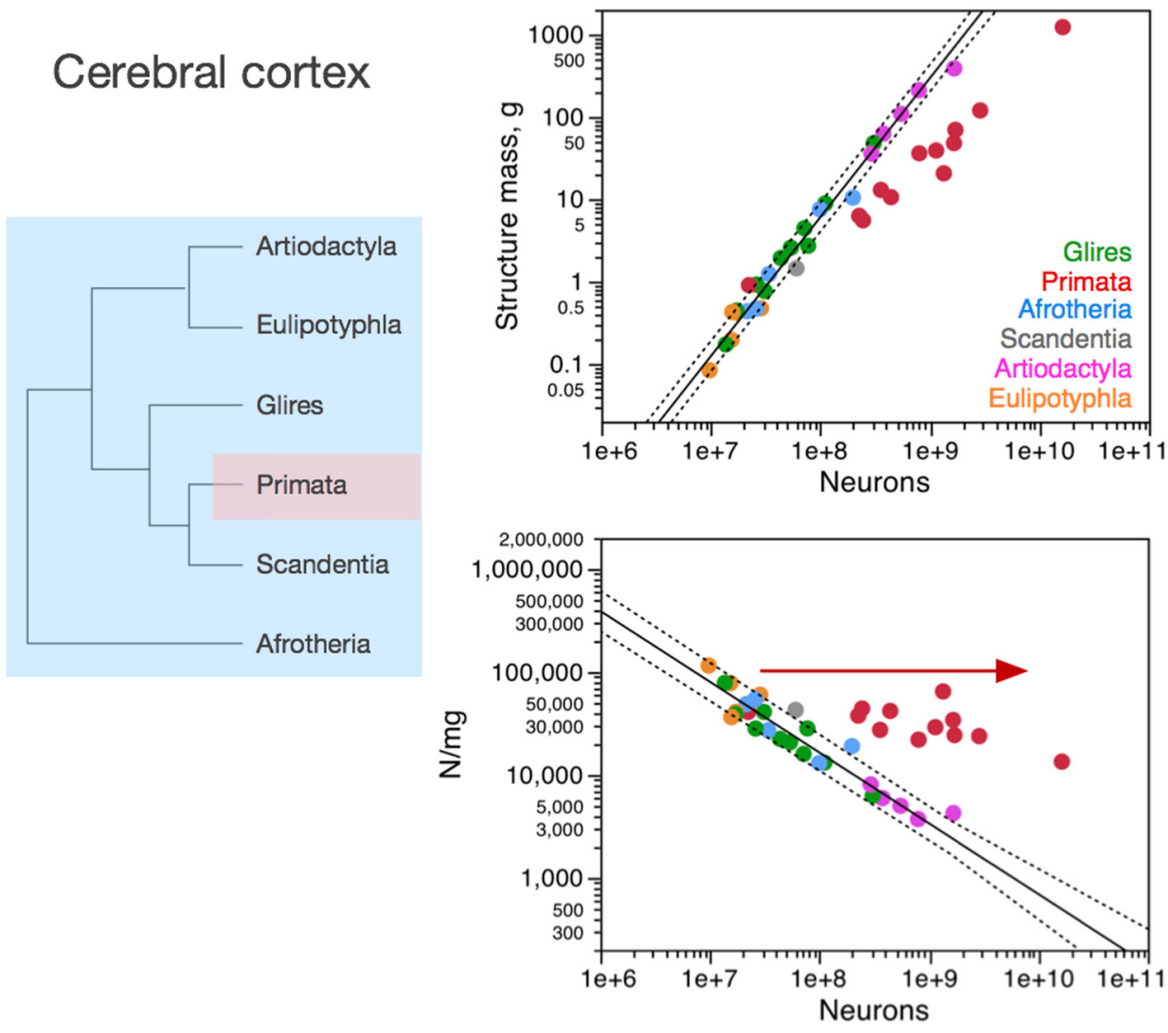
**Neuronal scaling rules for the cerebral cortex, that is, the relationship between cortical mass and number of neuronal cells, differs between primates and non-primates, but is shared across all non-primate species examined**. **Top right:** scaling of cerebral cortical mass (gray and white matter combined) as a function of numbers of neurons in the structure across species; **Bottom right:** scaling of neuronal density as a function of numbers of neurons in the structure. Notice that neuronal density decreases uniformly across species as the cerebral cortex gains neurons, except in primates, which we suggest that branched off the mammalian ancestor (to which the same rules shared by current non-primates applied) when a modification nearly stopped average neuronal cell size from increasing (and thus, neuronal density from decreasing) as the cortex gained neurons (red arrow). **Top**: primates, function (not plotted for clarity) has exponent 1.087 ± 0.074; all others, joint power function plotted has exponent of 1.688 ± 0.051. **Bottom**: Primates, exponent −0.150 ± 0.064 (not plotted for clarity); non-primates, exponent −0.688 ± 0.052. Each symbol represents the average values for the cerebral cortex in one species (afrotherians, blue; glires, green; eulipotyphlans, orange; primates, red; scandentia, gray; artiodactyls, pink). The phylogenetic scheme on the left indicates the clades that share the same neuronal scaling rules for the cerebral cortex, and the presumed extension of these shared scaling rules to the common ancestor to the non-primate clades while primates diverge from them.

**Figure 4:**
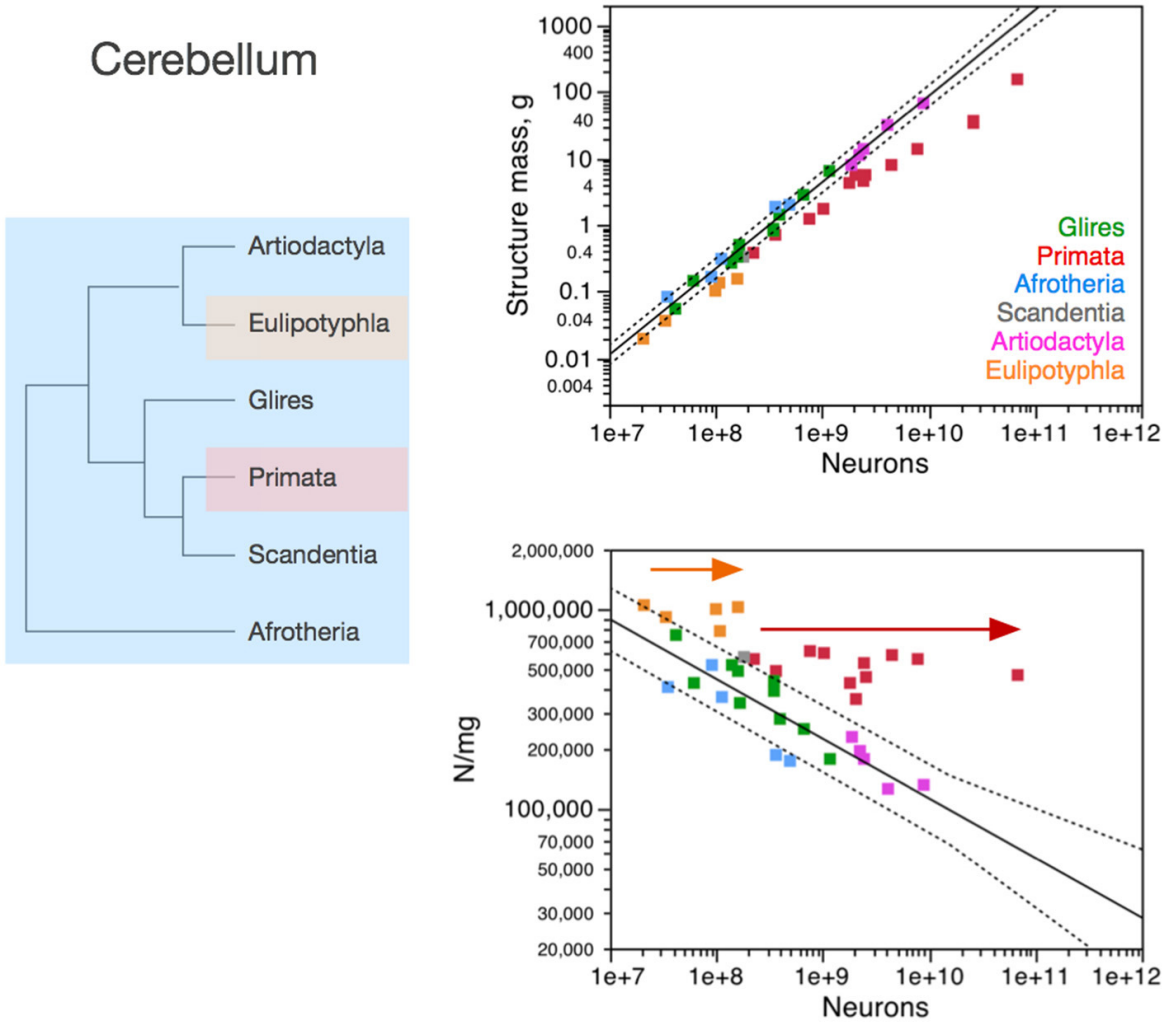
**Neuronal scaling rules for the cerebellum, that is, the relationship between cerebellar mass and number of neuronal cells, differs between primates, eulipotyphlans and other clades, but is shared across the latter**. **Top right:** scaling of cerebellar mass (gray and white matter combined) as a function of numbers of neurons in the structure across species. Non-primates, non-eulipotyphlans, joint exponent of 1.296 ± 0.043, *p* < 0.0001; primates, exponent of 0.976 ± 0.036, *p* < 0.0001; eulipotyphlans, exponent of 1.028 ± 0.084, *p* = 0.0012, not plotted for clarity. **Bottom right:** scaling of neuronal density as a function of numbers of neurons in the cerebellum. Non-primates, non-eulipotyphlans, joint exponent of −0.299 ± 0.046, *p* < 0.0001; primates and eulipotyphlans, *p* = 0.5822 and *p* = 0.7633, respectively. Notice that neuronal density decreases uniformly across species as the cerebellum gains neurons, except in primates and eulipotyphlans, which we suggest that branched off the mammalian ancestor with a modification that stopped average neuronal cell size in the cerebellum from increasing (and thus, neuronal density from decreasing) as the cerebellum gained neurons (orange and red arrows). Cerebellar neuronal density is higher in eulipotyphlans than in primates, indicating that these two groups do not share neuronal scaling rules for the cerebellum. Each symbol represents the average values for the cerebellum in one species (afrotherians, blue; glires, green; eulipotyphlans, orange; primates, red; scandentia, gray; artiodactyls, pink). The phylogenetic scheme on the left indicates in blue the clades that share the same neuronal scaling rules for the cerebellum, and the presumed extension of these shared scaling rules to the common ancestor to the non-primate, non-eulipotyphlan clades, while primates and eulipotyphlans diverge from them.

**Figure 5:**
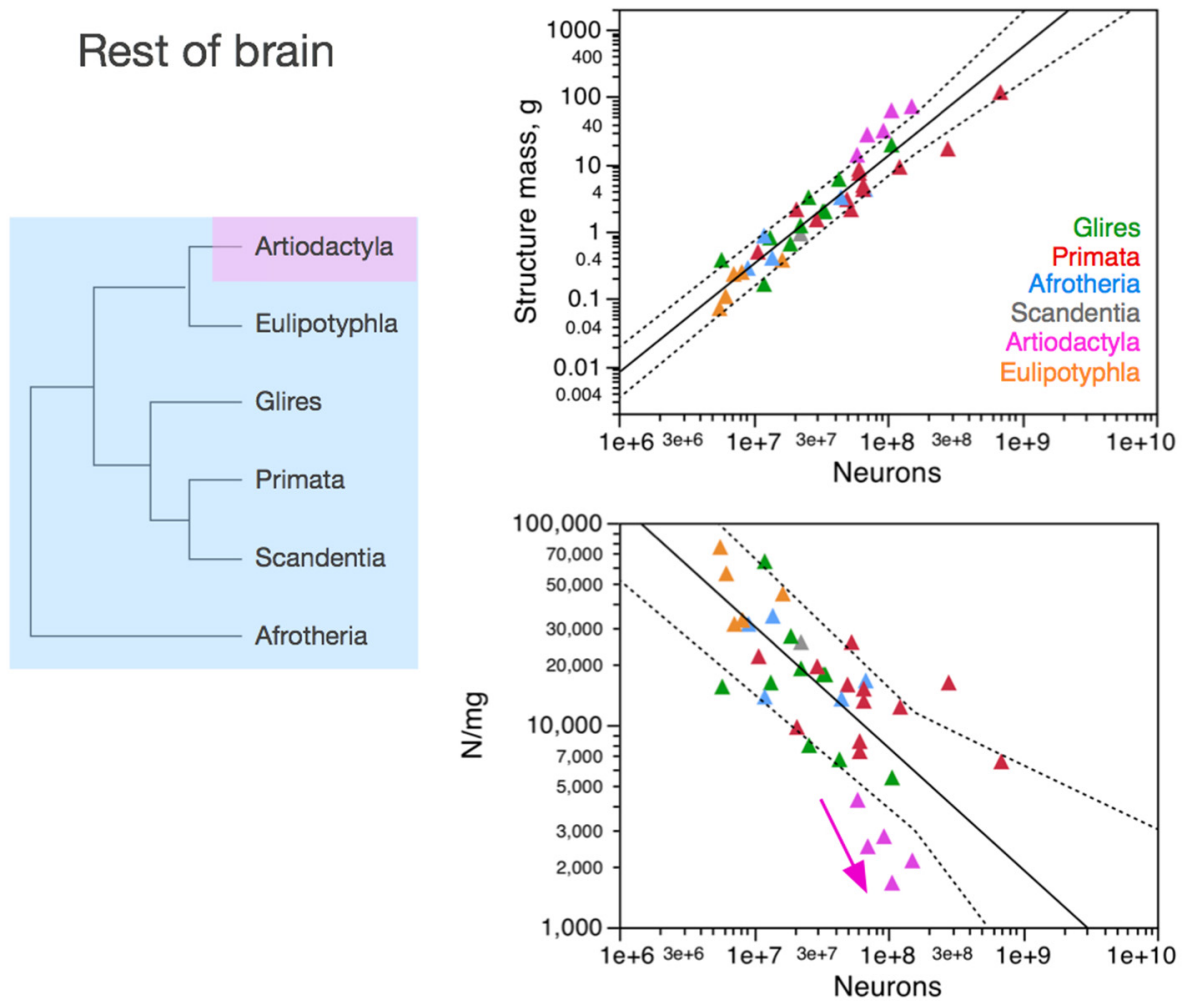
**Neuronal scaling rules for the rest of brain, that is, the relationship between rest of brain mass and number of neuronal cells, differs between artiodactyls and other clades, but is shared across all non-artiodactyl species examined**. **Top right:** scaling of rest of brain mass as a function of numbers of neurons in the structure across species. Plotted power function applies to all non-artiodactyls, with an exponent of 1.400 ± 0.077, *p* < 0.0001. **Bottom right:** scaling of neuronal density in the rest of brain as a function of numbers of neurons in the structure. Plotted power function applies to all non-artiodactyls, with exponent −0.398 ± 0.079, *p* < 0.0001. Notice that neuronal density decreases uniformly across species as the cerebral cortex gains neurons, but decreases even more steeply in artiodactyls (pink arrow), which we suggest that branched off the mammalian ancestor (to which the same rules shared by current non-artiodactyls applied) when a modification resulted in an even faster increase in average neuronal cell size (and thus, a faster decrease in neuronal density) as the rest of brain gained neurons (pink arrow). Each symbol represents the average values for the rest of brain in one species (afrotherians, blue; glires, green; eulipotyphlans, orange; primates, red; scandentia, gray; artiodactyls, pink). The phylogenetic scheme on the left indicates in blue the clades that share the same neuronal scaling rules for the rest of brain, and the presumed extension of these shared scaling rules to the common ancestor to the non-artiodactyl clades, while artiodactyls diverge from them (pink).

**Figure 6:**
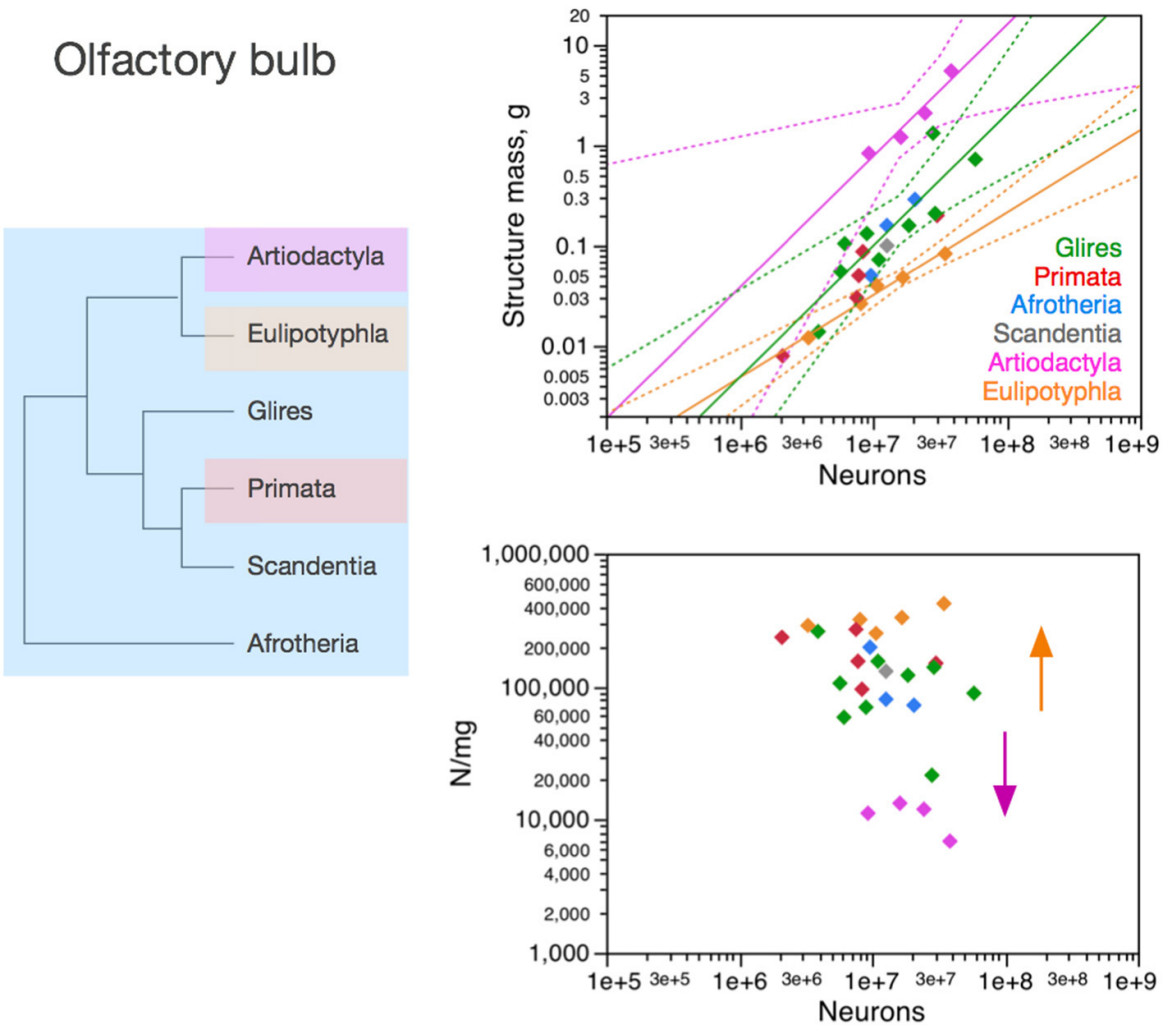
**Neuronal scaling rules for the olfactory bulb differ between eulipotyphlans, artiodactyls, primates and other clades**. **Top right:** scaling of olfactory bulb mass as a function of numbers of neurons in the structure across species. Plotted power functions have exponent of 0.823 ± 0.071, *p* = 0.0014 (eulipotyphlans, orange), 1.309 ± 0.257, *p* = 0.0364 (artiodactyls, pink), and 1.185 ± 0.186, *p* < 0.0001 (in green: scandentia, afrotherians and glires, excluding the capybara; Ribeiro et al., 2014). **Bottom right:** scaling of neuronal density in the olfactory bulb as a function of numbers of neurons in the structure. Power functions are not significant, but neuronal density is highest in eulipotyphlans and lowest in artiodactyls, which we suggest that branched off the mammalian ancestor when modifications resulted in decreased and increased average neuronal cell sizes, respectively (orange and pink arrows). Each symbol represents the average values for the rest of brain in one species (afrotherians, blue; glires, green; eulipotyphlans, orange; primates, red; scandentia, gray; artiodactyls, pink). The phylogenetic scheme on the left indicates in blue the clades that share the same neuronal scaling rules for the olfactory bulb, and the presumed extension of these shared scaling rules to the common ancestor to the non-artiodactyl clades, while artiodactyls and eulipotyphlans diverged from them.

Importantly, the relationships between brain structure mass and the number of neurons that compose the structure, that is, the neuronal scaling rules that apply to each brain structure, are not shared across all mammalian clades, but are also not exclusive of each clade. The neuronal scaling rules that apply to the cerebral cortex are shared by all clades analyzed here except primates (Figure 3); the neuronal scaling rules that apply to the cerebellum are shared by all clades except primates and eulipotyphlans (Figure 4); the neuronal scaling rules that apply to the rest of brain are shared by all (including primates) but exclude artiodactyls (Figure 5); and the neuronal scaling rules that apply to the olfactory bulb are shared only by afrotherians and glires, and not by eulipotyphlans, primates, or artiodactyls (Figure 6; Ribeiro et al., 2014). The exponents that apply to these relationships are given in the respective Figure legends.

The different relationships between structure mass and number of neurons across clades correspond to differential scaling of neuronal density (Figures 3–6, bottom right): any deviation from linearity in the relationship between number of neurons in a structure and the mass of this structure can be attributed to changes in the average mass of the cells in the structure. Given that other cell density changes very little and not systematically across mammalian species and orders (Figure 2), it can be inferred that changes in the neuronal scaling rules are mostly due to changes across clades in how the average mass of neurons in the structures scales as the structures gain neurons.

### Cerebral cortex

In the cerebral cortex (including paleo and archicortex, and thus not only neocortex), neuronal density decreases homogeneously with the addition of neurons across afrotherians, rodents and lagomorphs (glires), eulipotyphlans, scandentia and artiodactyls alike (Figure 3). Primates stand out by having much larger neuronal densities than other mammalian species for similar numbers of cortical neurons (Figure 3, bottom right). As a result, the primate cerebral cortex has more neurons than other mammalian cortices of similar size (mass). There still is a significant decrease in neuronal density that accompanies the addition of neurons to the cerebral cortex of primates, but it is much more subtle in primates than in the other clades (primates, −0.150 ± 0.064, *p* = 0.0416; all others, −0.688 ± 0.052, *p* < 0.0001).

The larger neuronal density and numbers of neurons in primate cortices compared to non-primate cortices is not due simply to a higher density of neurons in primate visual cortical area V1. Since this area holds about one third of all cortical neurons in non-human primates (Collins et al., 2013), if the deviation of primate cerebral cortex from non-primate neuronal scaling rules were due simply to unusual high neuronal densities in V1, then this deviation should be of the order of 30% compared to other species of similar cortical mass. Instead, monkeys have 3–5 more cortical neurons than rodents and artiodactyls of similar cortical mass. Moreover, large neuronal densities in primary visual cortex are not exclusive of primates: in the mouse cortex, area V1 is the functional area with the largest neuronal density, over 155,000 neurons/mm^3^, more than twice the density found in most other areas (Herculano-Houzel et al., 2013).

The homogeneous scaling of neuronal density in the cerebral cortex across modern non-primate species is a very strong suggestion that the neuronal scaling rules that apply to these crown species today have been conserved in their evolutionary history, and also applied to their ancestors as well as to the last common ancestor to all eutherians. Thus, considering that mammalian evolution originated with very small animals with very small brains and proceeded with a trend toward the addition of neurons, the homogeneous scaling of neuronal density in non-primate clades indicates that mammalian brain evolution, which mostly involved expansion of the cerebral cortex (Rowe et al., 2011), has occurred in non-primates with the addition of neurons to the cerebral cortex accompanied by a homogeneous increase in average neuronal cell size, according to the relationship shown in Figure 3. Evidence of a steep increase in neuronal cell size (more specifically, the size of dendritic arbors) in the cerebral cortex across rodent species with increasing cortical area (but far less in primates, as expected; see below) has just recently been provided (Elston and Manger, 2014).

We suggest that it was from these shared neuronal scaling rules that primates branched off. Remarkably, the smallest primates have high neuronal densities in the cerebral cortex that overlap with neuronal densities in the cortex of other mammals of similar mass or number of neurons (be they the closely related scandentia, or rodents, afrotherians, or eulipotyphlans; Figure 3). However, in the scenario we propose here, as the primate cortex gained neurons, its neuronal densities diverged more and more from neuronal densities in other clades. This overlap for the smallest cortices followed by divergence in neuronal densities in modern species suggests that the branching off of primates from the common ancestor with other mammalian clades happened with a change in the mechanisms that regulate neuronal cell size, such that average neuronal cell size no longer increased dramatically as the cortex gained neurons in the new animals. Indeed, recent evidence comparing rodent cortices to primate cortices confirms this hypothesis (Elston and Manger, 2014). Thus, the initial, small primates (as well as those who remained small in modern times) probably had cerebral cortices with high neuronal densities (that is, small neurons) that matched the neuronal density found in the cerebral cortex of modern non-primate mammals of a similar cortical mass. In contrast, primates with more cortical neurons probably benefited from having these neurons fit in a not-so-much-larger cortex compared to other mammals. This smaller cortical size is expected to carry the advantage of shorter conduction times than in larger cortices with similar numbers of neurons in non-primates. Indeed, we have estimated that propagation time increases much more steeply in rodents (with the number of cortical neurons raised to the power of 0.466) than in primates (with an exponent of 0.165; Mota and Herculano-Houzel, 2012). At the same time, we estimate the computational capacity of the white matter to scale faster with numbers of cortical neurons in primates than in rodents (Mota and Herculano-Houzel, 2012). We thus expect that genetic modifications related to primate-specific cortex evolution should be found in genes that control neuronal cell size and how neuronal cell size (including axonal arbors in the white matter) is tied to numbers of neuronal cells in the cerebral cortex, allowing numbers of neurons to increase in the absence of major increases in average neuronal cell size (soma, dendritic arbors, and axonal arborization).

### Cerebellum

In the cerebellum, neuronal density decreases homogeneously with the addition of neurons across afrotherians, rodents and lagomorphs (glires), and artiodactyls alike (Figure 4). In contrast, both primates and eulipotyphlans stand out by having much larger neuronal densities than other mammalian species for similar numbers of cerebellar neurons. As a result, the cerebellum of primates and eulipotyphlans has more neurons than other mammalian cerebella of similar mass. Given the shared neuronal scaling rules for the cerebellum among afrotherians, glires and artiodactyls, and the evolutionary relationships among these clades, we infer that these shared neuronal scaling rules are conserved and thus also applied to mammals prior to the divergence of primates and eulipotyphlans (Figure 4, left).

Notice that the neuronal scaling rules for the cerebellum are different between primates and eulipotyphlans: while cerebellar neuronal density scales with numbers of neurons neither in eulipotyphlans nor in primates (*p* > 0.5), it is higher in the former. For this reason, the evolutionary modifications in the cerebellar neuronal scaling rules that gave rise to these two groups were probably independent events in the origin of eulipotyphlans and in the origin of primates.

As in the cerebral cortex, the neuronal scaling rules for the cerebellum appear to have changed in the evolution of primates and eulipotyphlans with a modification in the relationship between average neuronal cell size and numbers of neurons. Similarly to the non-primate cerebral cortex, the non-primate, non-eulipotyphlan, and presumably ancestral cerebellum gained neurons with an accompanying significant increase in the average size of the neuronal cells, which translates into decreasing neuronal densities (exponent, −0.299 ± 0.046, *p* < 0.0001; Figure 4). Eulipotyphlans and primates of increasing cerebellar mass, however, have leveled-off neuronal densities (Figure 4, arrows), which, in the face of nearly constant non-neuronal densities, indicates that average neuronal size does not increase in the cerebellum of these species as it gains neurons in evolutionary history. Thus, we suggest that the divergence of primates from other mammals happened with a change in the mechanisms that regulate neuronal cell size in the primate cerebellum (as well as in the cerebral cortex), such that average neuronal cell size no longer increased dramatically as the cerebellum gained neurons in the new animals—and a similar type of modification occurred in the cerebellum, independently, in the branching off of eulipotyphlans from other mammals.

Fundamental to the interpretation of these findings is the recent realization from molecular phylogenetic studies that eulipotyphlans, which used to be considered as part of Insectivora, presumably the most basal mammals, are actually a more recent monophyletic clade, placed in Laurasitheria, next to bats, carnivores and cetartiodactyls (Madsen et al., 2001; Murphy et al., 2001, 2004). Thus, the scaling rules that apply to eulipotyphlan species alone cannot be considered to reflect an ancestral state. Rather, it is the rules that are shared by afrotherians (the most basal group in the eutherian evolutionary tree; Murphy et al., 2001, 2004) and other clades that can be inferred to also have applied to the common ancestor to all eutherians.

### Rest of brain

In contrast to the cerebral cortex and cerebellum, neuronal density in the “rest of brain” (that is, in the ensemble of brain tissue from brainstem to the striatum) decreases homogeneously with the addition of neurons across afrotherians, rodents and lagomorphs (glires), eulipotyphlans and primates alike—while artiodactyls stand out as outliers to the scaling rule that applies to all other species (Figure 5). Artiodactyls stand out by having much smaller, not larger, neuronal densities than other mammalian species for similar numbers of neurons in the rest of brain (Figure 5, bottom right). As a result, the non-cortical, non-cerebellar structures have *fewer* neurons in artiodactyls than in other mammalian species with a similar mass in the rest of brain (Figure 5, top right).

Although the scatter is much larger than found in the neuronal scaling rules for the cerebral cortex and cerebellum, the shared neuronal scaling rules for the rest of brain among afrotherians, glires, primates, scandentia, and eulipotyphlans allow us to infer, given the evolutionary relationships among these clades, that these shared neuronal scaling rules are evolutionarily conserved and also applied to mammals prior to the divergence of artiodactyls.

Contrary to the cerebral cortex of primates and the cerebellum of primates and eulipotyphlans, the neuronal scaling rules for the rest of brain in artiodactyls appear to have changed with a modification in the relationship between average neuronal cell size and numbers of neurons that resulted in an even *steeper* increase in the average size of the neuronal cells in the rest of brain, which translates into a steeper decrease in neuronal densities as the rest of brain gains neurons (Figure 5, bottom right). This would result, for instance, if axons became much wider in the rest of brain of artiodactyls than in other species with similar numbers of neurons in the rest of brain, and/or if more of these neurons were connected through long-distance fibers, both of which would lead to an increase in the proportion of white matter in the rest of brain of artiodactyls compared to other mammals with similar numbers of neurons, and thus presumably in neurons that are on average larger as a whole (including soma, dendrites, and axons) in artiodactyls than in other clades.

### Olfactory bulb

The olfactory bulb is an evolutionarily ancient structure in the brain, similarly to the brainstem, diencephalon and striatum, and as such it could be expected to be mostly conserved across mammalian clades, similarly to the rest of brain. In contrast, as we have shown recently, the neuronal scaling rules that apply to the olfactory bulb differ across eulipotyphlans (exponent, 0.823 ± 0.071, *p* = 0.0014), primates and glires (linear scaling; Ribeiro et al., 2014), and also artiodactyls (also linear, but excluding glires and primates; Figure 6, top right). In eulipotyphlans, the olfactory bulb has more neurons than in glires or primates with a similar olfactory bulb mass (Figure 6, orange); in contrast, in artiodactyls, similar numbers of neurons form an olfactory bulb that is nearly 10 times larger than in glires and primates, and even larger than in eulipotyphlans, although numbers of neurons in the olfactory bulb fall in the same range in all clades examined, including primates (Figure 6, top, pink).

While there is no obvious systematic scaling of neuronal density in the olfactory bulb in glires, rodents, primates and artiodactyls (all values of *p* > 0.2), eulipotyphlan olfactory bulbs clearly have higher neuronal densities, and artiodactyl olfactory bulbs have much lower neuronal densities, than other clades (Figure 6, bottom). Thus, our data suggest that the ancestral mammalian brain had an olfactory bulb composed of a number of neurons that conformed to the same scaling rules that still apply to modern afrotherians, glires, primates and scandentia, while eulipotyphlans branched off with a modification that led to a smaller average neuronal size in the olfactory bulb (and thus higher neuronal densities), and artiodactyls branched off with a modification in the other direction, that led to larger average neuronal size in the olfactory bulb (and rest of brain).

## Scaling of neuronal density across structures: coordinated changes in neuronal cell size

The analysis of neuronal scaling rules in the light of the phylogenetic relationships among the clades that share them or not, shown above, suggests the existence of a common set of scaling rules that apply to modern afrotherians, glires and artiodactyls (with the exception of the rest of brain), and thus can be inferred to also have applied to the common eutherian ancestor. From these neuronal scaling rules, we propose that primates branched off upon modifications that prevented average neuronal size from increasing (and thus neuronal density from decreasing) with the addition of neurons to the cerebral cortex and to the cerebellum; that eulipotyphlans branched off with modifications that prevented average neuronal size from increasing with the addition of neurons to the cerebellum; and artiodactyls branched off with modifications that led to an even steeper increase in average neuronal size in the rest of brain as it gained neurons.

Correlations between neuronal density and numbers of neurons in the different structures indicate that average neuronal cell size varies accompanying increases and decreases in numbers of neurons in each structure. This link between average neuronal cell size and numbers of neurons already provides an insight into the developmental mechanisms that lead to brains of different sizes: there must be pathways in place that tie the regulation of cell size (that is, soma plus dendritic and axonal arborizations) to proliferation of neuronal progenitors. Still, average neuronal cell size could in principle be regulated independently across different brain structures.

**Figure 7:**
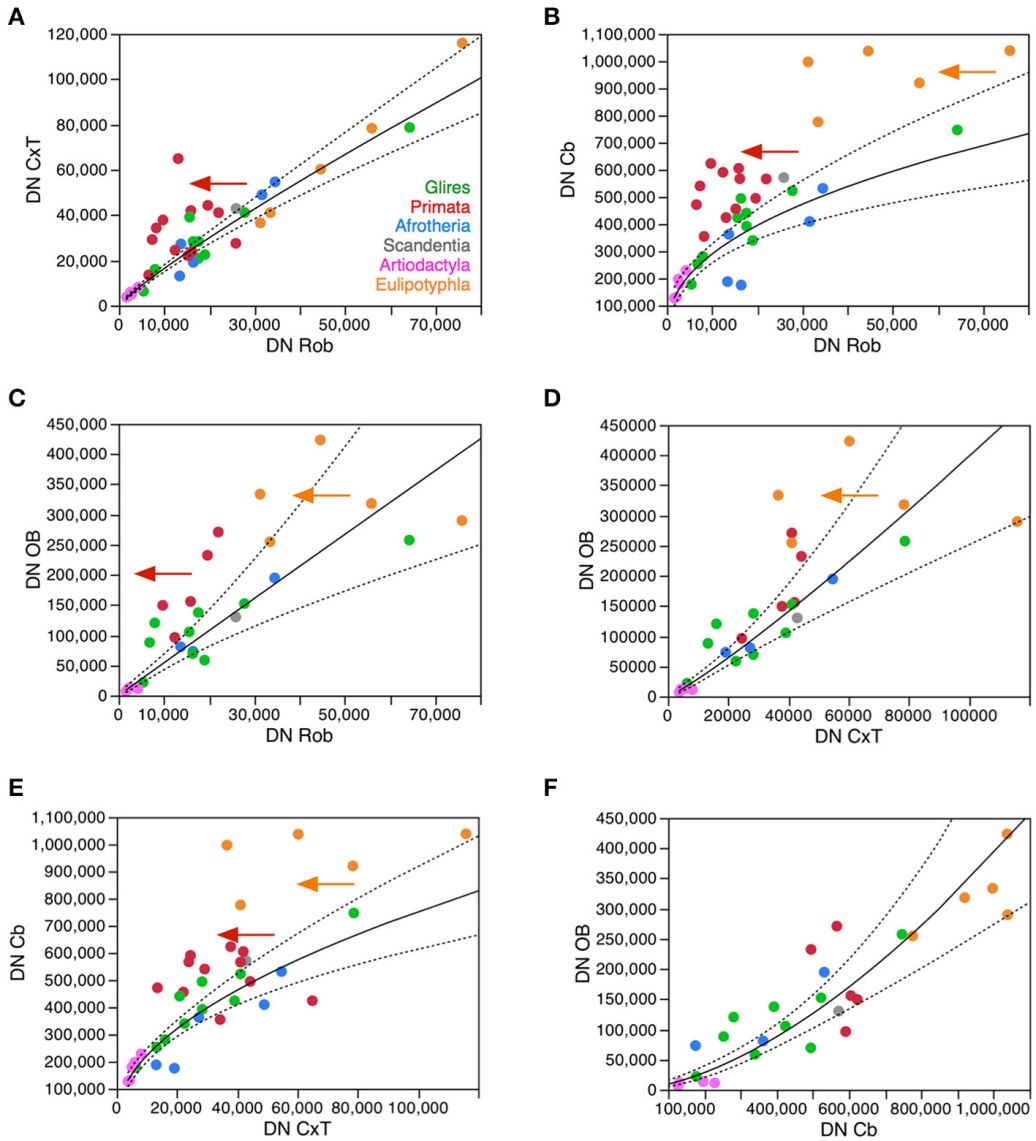
**Neuronal density varies concertedly between brain structures across species in most clades, but diverges in others**. Plots show how neuronal densities in general vary concertedly across species between the cerebral cortex and rest of brain **(A)**, between the cerebellum and rest of brain **(B)**, between the olfactory bulb and rest of brain **(C)**, between the olfactory bulb and the cerebral cortex **(D)**, between the cerebellum and the cortex **(E)**, and between the olfactory bulb and cerebellum **(F)**. **(A)** Plotted function excludes primates (red), and has exponent 0.872 ± 0.041 (*p* < 0.0001). **(B)** Plotted function excludes primates (red) and eulipotyphlans (orange), with an exponent of 0.446 ± 0.058, *p* < 0.0001. **(C)** Plotted function excludes primates (red) and eulipotyphlans (orange), with an exponent of 0.991 ± 0.011, *p* < 0.0001. **(D)** Plotted function excludes primates (red) and eulipotyphlans (orange), and has an exponent of 1.133 ± 0.112, *p* < 0.0001. **(E)** Plotted function excludes primates (red) and eulipotyphlans (orange), with an exponent of 0.529 ± 0.050, *p* < 0.0001. **(F)** Plotted function includes all clades, with an exponent of 1.630 ± 0.166, *p* < 0.0001. Each symbol represents the average values for the rest of brain in one species (afrotherians, blue; glires, green; eulipotyphlans, orange; primates, red; scandentia, gray; artiodactyls, pink).

Surprisingly, the analysis of variations in neuronal density across structures and species shows marked correlations, from which the primate and eulipotyphlan cerebellum and the primate cerebral cortex deviate as expected from the scenario described above. Neuronal densities in the cerebral cortex correlate uniformly with neuronal densities in the rest of brain across non-primate species, but primates have much higher neuronal densities in the cerebral cortex for the neuronal densities in their rest of brain compared to the other clades (Figure 7A). Neuronal densities in the cerebellum also correlate uniformly with neuronal densities in the rest of brain across non-primate, non-eulipotyphlan species, but primates and eulipotyphlans have much higher neuronal densities in the cerebellum for the neuronal densities in their rest of brain compared to the other clades (Figure 7B). Consistently, neuronal densities are correlated between the cerebellum and the cerebral cortex in the non-primate, non-eulipotyphlan species, while primates and eulipotyphlans have higher neuronal densities in the cerebellum than predicted from the neuronal densities in their cerebral cortices (Figure 7E). Neuronal densities are also correlated between the olfactory bulb and the rest of brain, cerebral cortex, and cerebellum (Figures 7C,D,F), although neuronal densities in the primate olfactory bulb are higher than predicted from the densities in their rest of brain (Figure 7C). Notice that, although neuronal densities are strongly correlated across all structures, they vary with different power exponents across structures (Figure 7). This implies that as one part of the brain gains somewhat larger neurons, neurons in different structures also become larger—but at different rates in different structures. As a consequence, there is no consistent relationship between total brain mass and neuronal densities in particular brain structures, although the mass of each structure is consistently associated with a predictable neuronal density as shown in Figures 3–6.

These findings show that, with few exceptions such as the primate cerebral cortex and primate and the eulipotyphlan cerebellum, increases in average neuronal cell size in one structure (and thus decreases in neuronal density) tend to be accompanied by increases in average neuronal cell size (and thus decreases in neuronal density) in other structures. This previously undescribed covariation across species between neuronal densities in different parts of the brain suggests that similar mechanisms influence average neuronal cell size in different structures of the same brain, such that when these mechanisms lead to increased average neuronal size in one structure accompanying increases in numbers of neurons, they lead to coordinatedly increased average neuronal size in other structures (although at different rates in different structures). As shown above, these deviations from the overall pattern do not stem simply from increased numbers of neurons in the primate cerebral cortex or in the primate and eulipotyphlan cerebellum relative to the number of neurons in the rest of brain (although these do occur; see below), for the neuronal densities in these structures deviate from the predicted given the numbers of neurons in these structures (Figures 3, 4). Rather, these deviations most likely reflect evolutionary modifications away from the concerted variation in neuronal density across structures shown in Figure 7. This concerted variation is however not linear, as also shown in Figure 7: neuronal density varies in concert across structures and species, but at different rates across different structures (see figure legend). A non-linear concerted variation in neuronal densities across structures however is still compatible with the existence of common mechanisms that influence average neuronal size throughout the brain as the different structures gain neurons. We propose that the primate cerebral cortex and the primate and eulipotyphlan cerebella diverged from these concerted relationships, branching off with modifications that allowed average neuronal cell size in these structures not to increase accompanying increases in average neuronal cell size in the rest of brain (Figures 7A,B), and also allowing a departure in the relationship between average neuronal cell size in the cerebral cortex and cerebellum from the relationship that supposedly applied to the common ancestor and still applies to modern afrotherians, glires and artiodactyls (Figure 7E).

## Scaling of numbers of neurons and average neuronal cell mass across structures

The concept of concerted scaling of brain structure size (volume) across species, called linked regularities by the authors who first described them (Finlay and Darlington, 1995), has been influential in comparative and evolutionary neuroanatomy. Since then, however, several analyses have shown that, at the same time as regularities do exist, brain structures are also relatively free to vary in relative size across clades, rather than exhibiting purely a single, homogeneous scaling of relative volume across species as brain volume varies (e.g., Barton and Harvey, 2000; Clark et al., 2001; Smaers and Soligo, 2013).

Volume, however, is not a meaningful parameter for inferring computational capacity of brains and their structures unless it is shown to serve as a proxy for presumably computational key features of brain structures such as numbers of neurons and numbers of synapses integrated by these neurons—and, as we show here (Figures 3–6), brain structure volumes do not vary homogeneously with numbers of neurons across structures and species. Volume (or mass) of brain structures is also a result of numbers of cells and their average volume, and not a factor that determines cell numbers and size. Numbers of neurons, thus, must be compared directly across structures to determine whether they indeed scale regularly and in a linked manner between brain structures across mammalian clades.

**Figure 8:**
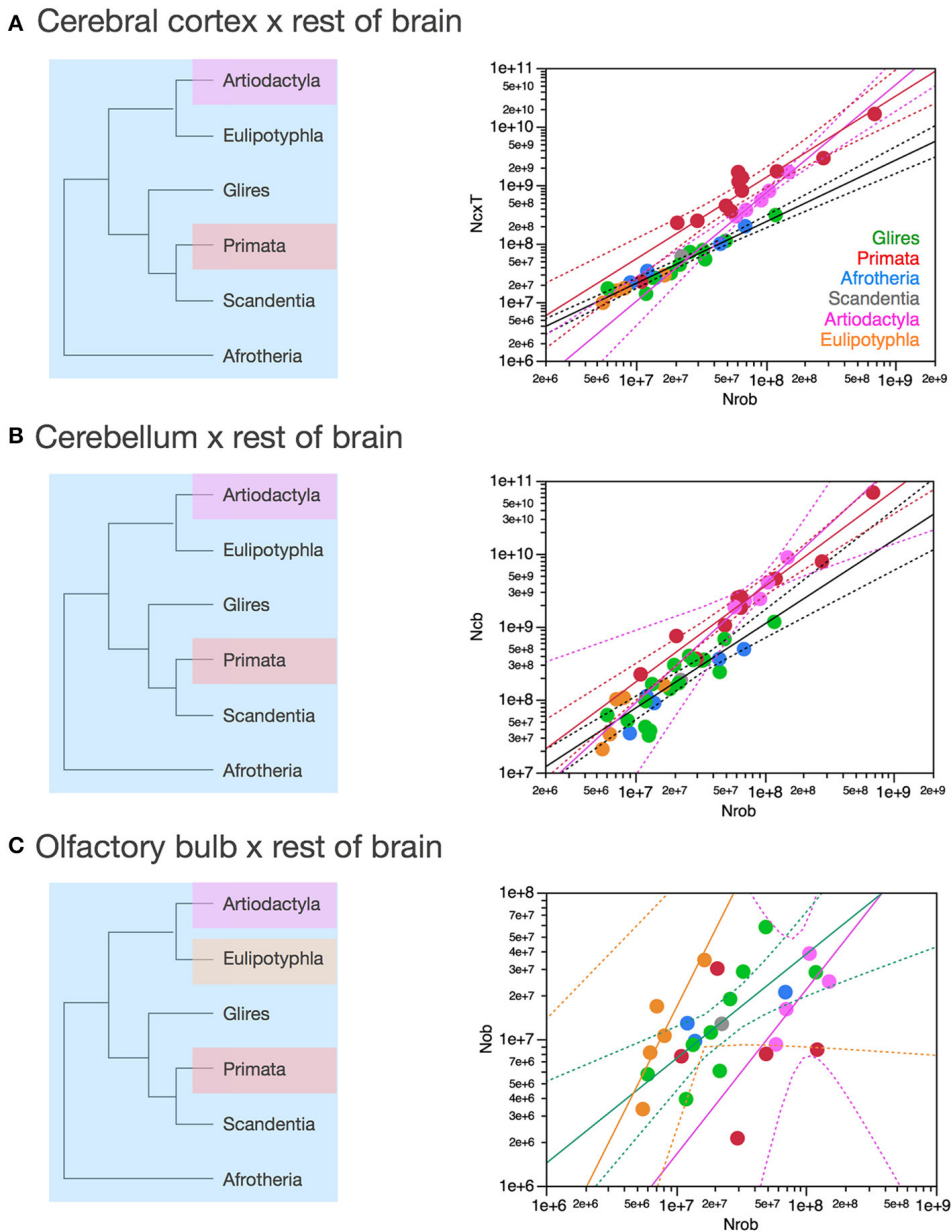
**Scaling of numbers of neurons in the cerebral cortex, cerebellum and olfactory bulb as a function of numbers of neurons in the rest of brain varies across clades**. Each symbol represents the average values for the structures indicated in one species (afrotherians, blue; glires, green; eulipotyphlans, orange; primates, red; scandentia, gray; artiodactyls, pink). The phylogenetic scheme on the left indicates in blue the clades that share the same neuronal scaling rules for the rest of brain, and the presumed extension of these shared scaling rules to the common eutherian ancestor; clades that have divergent scaling rules are colored differently. **(A)** Scaling of numbers of neurons in the cerebral cortex as a function of numbers of neurons in the rest of brain across species. Power functions plotted have exponents 1.391 ± 0.158, *p* < 0.0001 (primates, in red), 1.852 ± 0.135, *p* = 0.0008 (artiodactyls, in pink), and 1.053 ± 0.061, *p* < 0.0001 (afrotherians, glires, scandentia and eulipotyphlans, in black). **(B)** Scaling of numbers of neurons in the cerebellum as a function of numbers of neurons in the rest of brain across species. Power functions plotted have exponents 1.315 ± 0.112, *p* < 0.0001 (primates, in red), 1.632 ± 0.222, *p* = 0.0148 (artiodactyls, in pink), and 1.154 ± 0.112, *p* < 0.0001 (afrotherians, glires, scandentia and eulipotyphlans, in black). **(C)** Scaling of numbers of neurons in the olfactory bulb as a function of numbers of neurons in the rest of brain across species. Power functions plotted have exponents 1.770 ± 0.578, *p* = 0.0548 (eulipotyphlans, in orange), 1.127 ± 0.638, *p* = 0.2194 (artiodactyls, in pink), and 0.714 ± 0.181, *p* = 0.0023 (afrotherians, glires, and scandentia, in black).

Figure 8 shows that they do not: even across gross brain regions, such as the entire cerebral cortex, the entire cerebellum and the ensemble of brainstem, diencephalon and striatum (the “rest of brain”), the rate at which one structure gains neurons as another also gains neurons varies across clades. The cerebral cortex gains neurons as a linear function of numbers of neurons in the rest of brain that is shared across afrotherians, glires, scandentia and eulipotyphlans, with exponent 1.053 ± 0.061 (*p* < 0.0001, *r*^2^ = 0.939; Figure 8A, red). Artiodactyls, on the other, gain neurons in the cerebral cortex faster than they gain neurons in the rest of brain, as a power function of exponent 1.391 ± 0.158 (*p* < 0.0001, *r*^2^ = 0.885), and also have more neurons in the cerebral cortex than glires with similar numbers of neurons in the rest of brain, falling well outside of the 95% confidence interval that applies to the ensemble of afrotherians, glires, and scandentia (Figure 8A, pink). Primates on the other hand, gain neurons in the cerebral cortex at an even steeper rate as a function of neurons added to the rest of brain, with exponent 1.852 ± 0.135 (*p* < 0.0001, *r*^2^ = 0.984; Figure 8A, pink). The discrepancy between primates, artiodactyls, and the ensemble of other clades suggests that the former two clades diverged from the common ancestor with modifications that generated larger numbers of neurons in the cerebral cortex than in the rest of brain, that is, with an actual relative expansion of the neuronal population in the cerebral cortex over the brainstem, diencephalon and striatum.

The cerebellum also gains neurons relative to the rest of brain at different rates between primates and artiodactyls compared to other mammals. The cerebellum gains neurons as a function of numbers of neurons in the rest of brain that is not significantly different from linearity (exponent 1.154 ± 0.112, *p* < 0.0001, *r*^2^ = 0.849) across afrotherians, glires, scandentia, and eulipotyphlans (Figure 8B, black). Artiodactyls, in contrast, gain neurons in the cerebellum faster than they gain neurons in the rest of brain, as a power function of exponent 1.632 ± 0.322 (*p* < 0.0001, *r*^2^ = 0.895; Figure 8B, pink), and so do primates, with an exponent of 1.315 ± 0.112 (*p* < 0.0001, *r*^2^ = 0.939; Figure 8B, red). The exponents for artiodactyls and primates are not significantly different, and numbers of neurons in the cerebellum are similar in the two clades for similar numbers of neurons in the rest of brain, although all fall well outside of the 95% confidence interval that applies to the ensemble of afrotherians, glires, eulipotyphlans, and scandentia (Figure 8B, black). The discrepancy between primates, artiodactyls, and the ensemble of other clades suggests that, as for the cerebral cortex, the former two clades diverged from the common ancestor with modifications that generated larger numbers of neurons in the cerebellum than in the rest of brain, that is, with an actual relative expansion of the neuronal population in the cerebellum over the rest of brain (Figure 8B, left).

**Figure 9:**
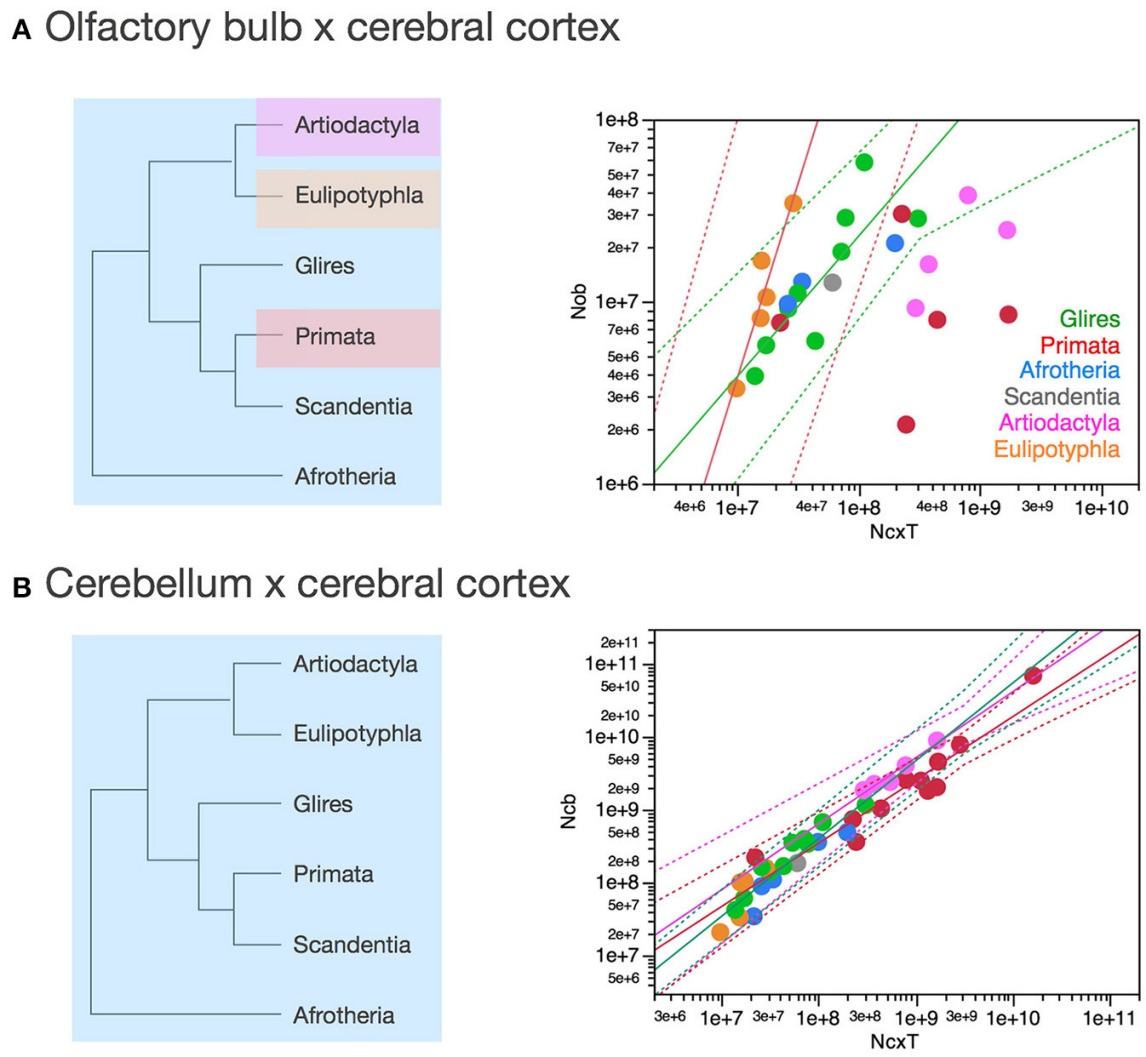
**Scaling of numbers of neurons in the olfactory bulb as a function of numbers of neurons in the cerebral cortex varies across clades, while numbers of neurons in the cerebellum vary coordinately with numbers of neurons in the cerebral cortex across all clades**. Each symbol represents the average values for the structures indicated in one species (afrotherians, blue; glires, green; eulipotyphlans, orange; primates, red; scandentia, gray; artiodactyls, pink). **(A)** Scaling of numbers of neurons in the olfactory bulb as a function of numbers of neurons in the cerebral cortex across species. Power functions plotted have exponents 2.129 ± 0.428, *p* = 0.0156 (eulipotyphlans, in orange), and 0.771 ± 0.188, *p* = 0.0046 (afrotherians, glires and scandentia, in green). The phylogenetic scheme on the left indicates in blue the clades that share the same neuronal scaling rules for the allocation of neurons in the olfactory bulb relative to the cerebral cortex, and the clades that have diverged from the presumed ancestral scaling rules (artiodactyls, eulipotyphlans, and primates). Primates are considered to also diverge from the ancestral scaling rules given their non-conformity to the relationship that applies jointly to afrotherians, glires, and scandentia. **(B)** Scaling of numbers of neurons in the cerebellum as a function of numbers of neurons in the cerebral cortex across species. The phylogenetic scheme on the left indicates in blue that all clades share similar neuronal scaling rules for the allocation of neurons in the cerebellum relative to the cerebral cortex. Power functions plotted are overlapping and have exponents 0.867 ± 0.108, *p* < 0.0001 (primates, in red), 0.904 ± 0.110, *p* = 0.0038 (artiodactyls, in pink), and 1.066 ± 0.111, *p* < 0.0001 (afrotherians, glires and scandentia, in green). The ensemble of species can be fitted by a linear function of slope 4.12 (*p* < 0.0001, not plotted).

The olfactory bulb gains neurons relative to the rest of brain at a rate that appears much faster in eulipotyphlans (exponent, 1.770 ± 0.578, *p* = 0.0548) than in the ensemble of afrotherians, glires, and scandentia (exponent, 0.714 ± 0.181, *p* = 0.0023), although the exponent does not reach significance in eulipotyphlans (Figure 8C). However, the olfactory bulb gains neurons at a significantly greater rate than the cerebral cortex in eulipotyphlans, as we have noted recently, as a power function of numbers of neurons in the cerebral cortex of exponent 2.129 ± 0.428 (*p* = 0.0156, *r*^2^ = 0.892; Figure 9A; Ribeiro et al., 2014), which suggests that, despite the statistical uncertainty, the eulipotyphlan olfactory bulb also gains neurons faster than the rest of brain. Afrotherians, glires and scandentia, in contrast, gain neurons more slowly in the olfactory bulb than in the cerebral cortex (exponent, 0.771 ± 0.188, *p* = 0.0046; Figure 9A), and the shared scaling across these clades suggests that this was the scaling rule that applied to ancestral mammals. Artiodactyls, primates and eulipotyphlans in turn diverged from the ancestral scaling rules with changes in the rate at which neurons are added to the olfactory bulb relative to both the rest of brain (Figure 8C) and to the cerebral cortex (Figure 9A), such that in eulipotyphlans these rates are greatly increased, but in artiodactyls and primates, numbers of neurons in the olfactory bulb are uncoupled from numbers of neurons in the cerebral cortex (and in the rest of brain).

In contrast, the cerebellum and the cerebral cortex gain neurons at approximately the same rate across clades as the rest of brain gains neurons. The direct comparison across the two structures shows that the number of neurons in the cerebellum varies as power functions of the number of neurons in the cerebral cortex with similar exponents across clades, all indistinguishable from linearity (artiodactyls, 0.904 ± 0.110; primates, 0.867 ± 0.108; glires, afrotherians and scandentia, 1.066 ± 0.111; only for eulipotyphlans the exponent does not reach significance, with *p* = 0.0623; Figure 9B). The relationship for the ensemble of clades can also be fit with a linear function of slope 4.12 (*p* < 0.0001, *r*^2^ = 0.985). Artiodactyls and afrotherians thus also conform to the linear scaling of neurons between cerebellum and cerebral cortex that we had shown previously to apply to rodents, primates and eulipotyphlans, with on average 4 neurons added to the cerebellum for every neuron added to the cerebral cortex (Herculano-Houzel, 2010). However, more precise information can be glimpsed from the scaling of ratios of neurons across brain structures. This suggests that a coordinated, linear addition of neurons to the cerebral cortex and to the cerebellum, regardless of the ratios of numbers of neurons in these structures to the rest of brain, is a universal characteristic of extant mammalian brains, and thus also applied to the common ancestor of eutherians (Figure 9B, left).

## Scaling of ratios of neurons over the rest of brain

The spinal cord and brainstem are the portions of the central nervous system that are most directly related to the regulation of bodily functions, and thus could be expected to scale in close relationship to the scaling of body physiology in its various aspects. Neurons in the cerebral cortex and the cerebellum, in contrast, are believed to add a whole new level of elaboration to the processing of information relayed from the body and back to it through associative processing, endowing animals with more refined and flexible behavioral repertoires.

In the absence of data on numbers of neurons and volumetric data for the spinal cord, the ratio of cortical volume over the volume of the medulla has been proposed as a value that should predict cognitive capacity in a manner that is not biased by body mass (Passingham, 1975). Variations in this ratio across primate species indeed were well correlated with available behavioral data, but so were brain size, relative cortical volume (Passingham, 1975), and encephalization quotient (Jerison, 1973). However, that comparison assumed that the volumes of the cerebral cortex and of the medulla are good proxies for numbers of neurons in the structures, whereas we have shown that this is not the case across clades. Thus, the ratio between numbers of neurons in the cerebral cortex and in the brainstem, or spinal cord, might provide a good estimate of how cortical processing capacity scales beyond body-related information processing across species.

Across primate species, we found that numbers of neurons in the spinal cord are linearly related to the length of the spinal cord, not body mass (Burish et al., 2010). Remarkably, the cerebral cortex gains neurons as a power function of numbers of neurons in the spinal cord with exponent 2.112, even though the mass of the cerebral cortex (including the white matter) scales only slightly faster than the mass of the spinal cord, as a power function of exponent 1.124 (Burish et al., 2010).

At this point, unfortunately, data on total numbers of neurons in the spinal cord that can be compared to numbers of neurons in the brain are only available for primates (Burish et al., 2010). However, we found in that study that the number of neurons in the ensemble of brainstem, diencephalon and striatum, which we refer to as “rest of brain”, scales linearly with the number of neurons in the primate spinal cord (Burish et al., 2010). This linearity warrants the use of numbers of neurons in the rest of brain, which are available for all 41 species in our sample, as a proxy for numbers of neurons in the spinal cord and also for the increase in numbers of neurons that would be directly related to any variations in body size (regardless of whether total volume, sensory surface area, muscular mass or energetic requirement is the relevant parameter). We thus use numbers of neurons in these structures as an internal reference for the examination of how information processing might scale faster in the cerebral cortex and in the cerebellum than required for dealing strictly with bodily functions, without having body mass as a confounding variable.

### Relative expansion of numbers of neurons in the cerebral cortex over the rest of brain

**Figure 10:**
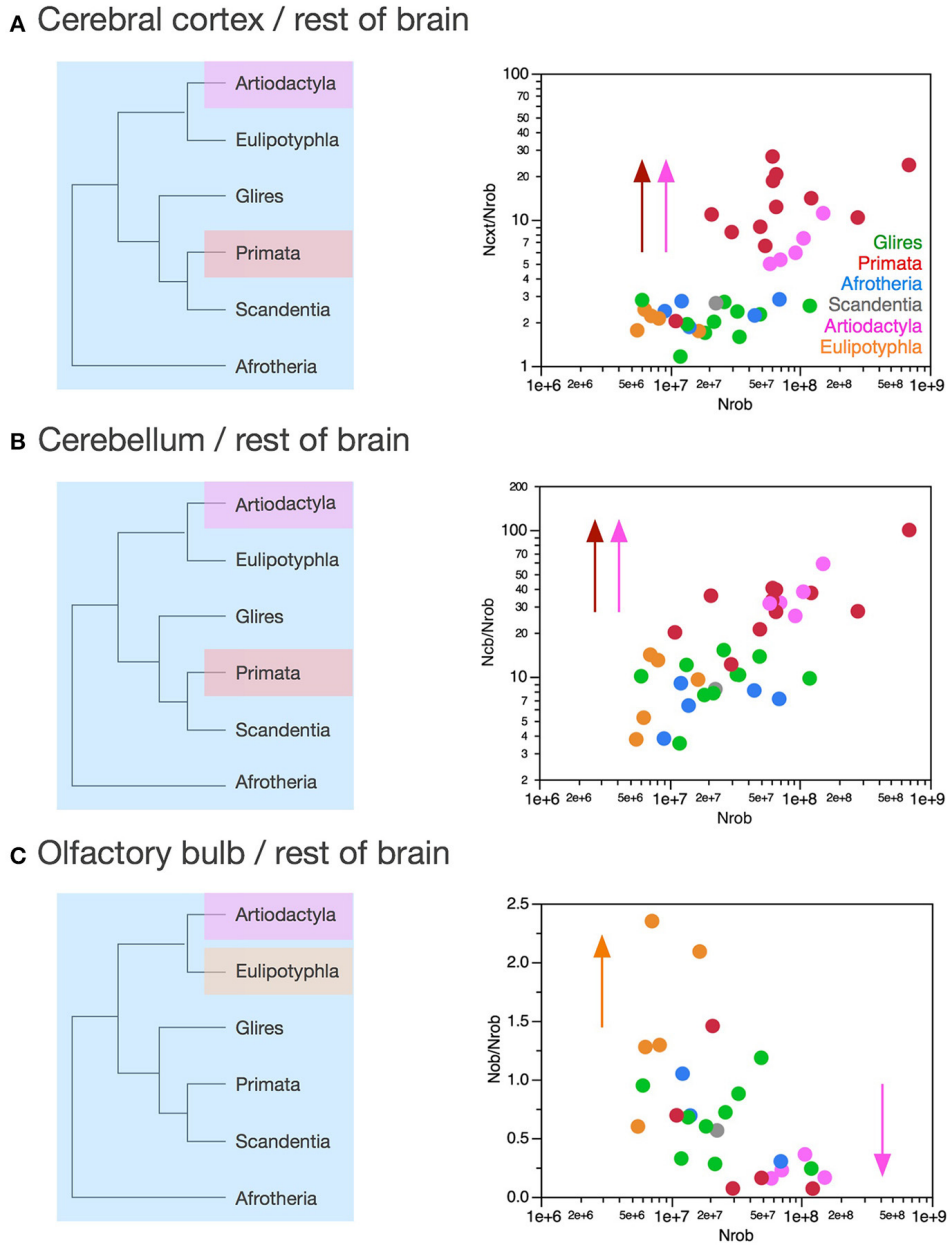
**Variation in the ratios between numbers of neurons in each structure and numbers of neurons in the rest of brain across clades show a relative increase in numbers of neurons in the cerebral cortex and cerebellum in both primates and artiodactyls**. Each symbol represents the average values for the structures indicated in one species (afrotherians, blue; glires, green; eulipotyphlans, orange; primates, red; scandentia, gray; artiodactyls, pink). **(A)** Ratio between numbers of neurons in the cerebral cortex and rest of brain is higher in primates (13.58 ± 2.14, red) and artiodactyls (6.96 ± 1.11, pink) than in afrotherians (2.41 ± 0.18, blue), glires (2.11 ± 0.17, green), eulipotyphlans (2.04 ± 0.13, orange) and scandentia (2.69, gray). The arrows and the phylogenetic scheme on the left indicate the divergence of primates and artiodactyls from the ratio shared by afrotherians, eulipotyphlans, scandentia and glires, and thus presumably also by ancestral mammals. **(B)** Ratio between numbers of neurons in the cerebellum and rest of brain is also higher in primates (35.91 ± 6.95, red) and artiodactyls (37.34 ± 5.72, pink) than in afrotherians (6.88 ± 0.89, blue), glires (10.03 ± 1.05, green), eulipotyphlans (9.14 ± 2.05, orange) and scandentia (8.24, gray). The arrows and the phylogenetic scheme on the left indicate the divergence of primates and artiodactyls from the ratio shared by afrotherians, eulipotyphlans, scandentia and glires, and thus presumably also by ancestral mammals. **(C)** ratio between numbers of neurons in the olfactory bulb and rest of brain are larger than 1 only in eulipotyphlans (1.52 ± 0.31, orange), compared to 0.65 ± 0.11 in glires, 0.68 ± 0.22 in afrotherians, 0.49 ± 0.27 in primates, 0.56 in scandentia, and 0.23 ± 0.05 in artiodactyls. The arrows and the phylogenetic scheme on the left indicate the divergence of eulipotyphlans and artiodactyls from the ratio shared by afrotherians, primates, scandentia and glires, and thus presumably also by ancestral mammals.

Afrotherians, glires, scandentia, and eulipotyphlans have on average 2.21 ± 0.22 neurons in the cerebral cortex to every neuron in the rest of brain, with ratios that are not significantly different across clades (ANOVA, *p* = 0.0783) and that do not vary in correlation with increasing numbers of neurons in the rest of brain (Spearman correlation, *p* > 0.5; Figure 10A). Thus, as the rest of brain gains neurons in these clades, the cerebral cortex does not gain relatively more neurons, maintaining a fairly stable ratio of approximately 2 neurons for every neuron in the rest of brain.

Artiodactyls, in contrast, have on average 6.96 ± 1.11 neurons in the cerebral cortex to every neuron in the rest of brain, which is a significantly larger ratio than in afrotherians, eulipotyphlans, glires and scandentia (Wilcoxon, *p* < 0.02; Figure 10A, pink). In contrast to those clades, the ratio between numbers of neurons in the artiodactyl cerebral cortex and in the rest of brain, N_CX_/N_ROB_, increases significantly with increasing numbers of neurons in the rest of brain (Spearman ρ = 1.000, *p* < 0.0001), as a power function of exponent 0.852 ± 0.135 (*p* = 0.0081, *r*^2^ = 0.930). In our sample, the artiodactyl cerebral cortex has between 5 (in the pig) and 11 (in the giraffe) times more neurons than the rest of brain, and the larger the brain (that is, the more the neurons in the rest of brain), the larger the N_CX_/N_ROB_.

Primates, with a N_CX_/N_ROB_ of 13.58 ± 2.14, have on average even more neurons in the cerebral cortex per neuron in the rest of brain than afrotherians, glires, eulipotyphlans and scandentia (ANOVA, *p* < 0.0001), although the primate N_CX_/N_ROB_ is not significantly different from the ratio in artiodactyls (Wilcoxon, *p* = 0.0512; Figure 10A, compare red and pink data points). The N_CX_/N_ROB_ ratio in primates increases significantly as the rest of brain gains neurons (Spearman ρ = 0.622, *p* = 0.0307), and as a power function of exponent 0.391 ± 0.158 (*p* = 0.0329, *r*^2^ = 0.380), significantly smaller than the exponent found in artiodactyls (Figure 10A). This means that the larger artiodactyl brains, with more neurons in the rest of brain, have N_CX_/N_ROB_ ratios that are comparable to those found in primates. Thus, while primates have on average ca. 7 times more neurons in the cerebral cortex relative to the rest of brain than afrotherians, glires, eulipotyphlans and scandentia, they overlap with several artiodactyl species. Of note, the human N_CX_/N_ROB_ ratio, at 23.68, is not the highest among primates: the bonnet monkey (*Macaca radiata*) has a higher N_CX_/N_ROB_ ratio of 27.05, and the much smaller squirrel monkey (*Saimiri sciureus*) brain has a fairly similar N_CX_/N_ROB_ of 20.45, while the smallest primate in our sample, the mouse lemur (*Microcebus murinus*), has an N_CX_/N_ROB_ of only 2.03, similar to the ratio found in other clades (Figure 10A).

The discrepancy in N_CX_/N_ROB_ rations between primates, artiodactyls, and the ensemble of other clades supports the conclusion that the former two clades diverged from the common ancestor with modifications that generated relatively larger numbers of neurons in the cerebral cortex than in the rest of brain, that is, with an actual relative expansion of the neuronal population in the cerebral cortex over the rest of brain (Figure 10A, left).

### Relative expansion of numbers of neurons in the cerebellum over the rest of brain

Similarly to N_CX_/N_ROB_, the ratio between numbers of neurons in the cerebellum and in the rest of brain, N_CB_/N_ROB_, is larger in artiodactyls (37.34 ± 5.72) and primates (35.91 ± 6.95) compared to afrotherians (6.88 ± 0.89), glires (10.03 ± 1.05), scandentia (8.24) and eulipotyphlans (9.14 ± 2.05; Figure 10B). Amongst these four latter clades, there is no significant difference in N_CB_/N_ROB_ (ANOVA, *p* = 0.3233). In none of these clades is the N_CB_/N_ROB_ ratio significantly correlated with the number of neurons in the rest of brain (Spearman, *p* > 0.05). Notably, in this case the human brain has the highest N_CB_/N_ROB_ ratio, at 100.04, well above artiodactyls (range, 26.04–58.94), while the next highest N_CB_/N_ROB_ ratios in primates overlap with ratios found in artiodactyls: the capuchin monkey (*Cebus apella*) has an N_CB_/N_ROB_ ratio of 40.26, and the long-tailed monkey (*Macaca fascicularis*) has an N_CB_/N_ROB_ of 39.27 (Figure 10A).

The N_CB_/N_ROB_ ratio is approximately 4 times larger than the N_CX_/N_ROB_ in afrotherians, glires, scandentia and eulipotyphlans, consistently with an average ratio of addition of 4.12 neurons to the cerebellum to every neuron added to the cerebral cortex reported above. In contrast, in primates, the N_CB_/N_ROB_ ratio is only about 3 times larger than the N_CX_/N_ROB_ ratio, while in artiodactyls, the average N_CB_/N_ROB_ ratio is 6 times larger than the N_CX_/N_ROB_ ratio. This suggests that while neurons increase linearly in numbers between the cerebellum and the cerebral cortex, the ratio between numbers of neurons in the two structures (that is, the slope of the linear relationship) might actually differ between clades.

### Relative allocation of numbers of neurons in the cerebellum and cerebral cortex

**Figure 11:**
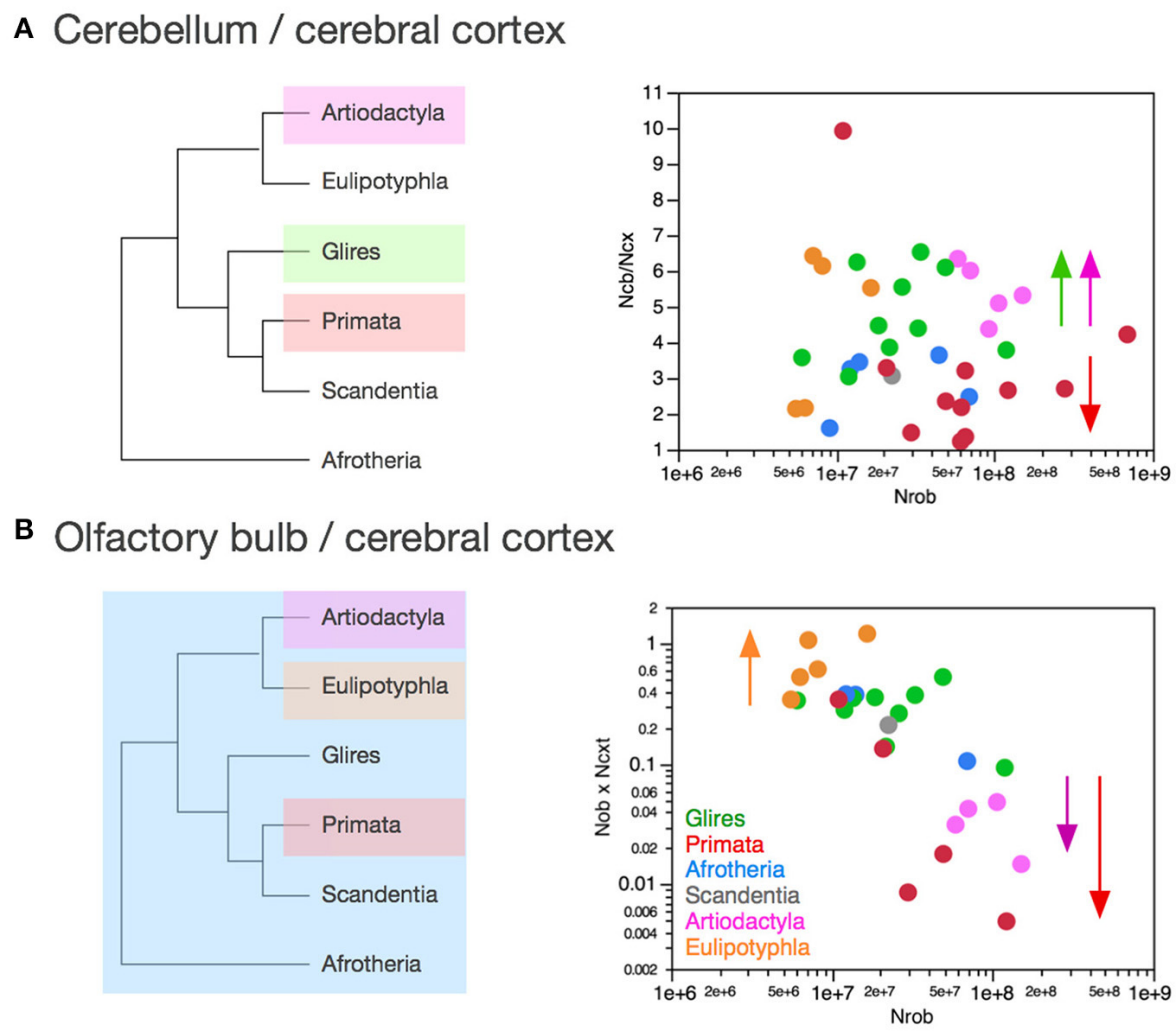
**Clade-specific ratios between numbers of neurons in the olfactory bulb and cerebral cortex, and between numbers of neurons in the cerebellum and cerebral cortex**. Each symbol represents the average values for the structures indicated in one species (afrotherians, blue; glires, green; eulipotyphlans, orange; primates, red; scandentia, gray; artiodactyls, pink). **(A)** Ratio between numbers of neurons in the cerebellum and cerebral cortex is higher in artiodactyls (5.43 ± 0.35), glires (4.76 ± 0.40, green) and eulipotyphlans (4.48 ± 0.96, orange) than in afrotherians (2.89 ± 0.38, blue), primates (3.15 ± 0.73) and scandentia (3.07, gray). The arrows indicate the differences cross clades, although the absence of clearly shared rates between at least afrotherians and glires precludes inferring the ratios that applied to ancestral mammals. **(B)** Ratio between numbers of neurons in the olfactory bulb and cerebral cortex is much higher in eulipotyphlans (0.75 ± 0.16, orange) than in all other clades (afrotherians, 0.29 ± 0.09; glires, 0.30 ± 0.04; scandentia, 0.21) and particularly low in artiodactyls (0.03 ± 0.01, pink). The arrows and the phylogenetic scheme on the left indicate the divergence of primates and artiodactyls from the ratio shared by afrotherians, eulipotyphlans, scandentia and glires, and thus presumably also by ancestral mammals.

As reported above, the scaling of numbers of neurons in the cerebellum as a function of the number of neurons in the cerebral cortex can be described as a linear relationship across the ensemble of all clades analyzed here with slope 4.12 (*p* < 0.0001, *r*^2^ = 0.985). Although the relationship within each clade is indistinguishable from linearity, the direct analysis of the ratio between numbers of neurons in the cerebellum and in the cerebral cortex shows a distinction across some clades (Figure 11A). Glires and artiodactyls have average ratios of neurons in the cerebellum relative to the cerebral cortex (N_CB_/N_CX_) of 4.76 ± 0.40 and 3.15 ± 0.73, respectively, which are significantly higher than the ratios found in afrotherians (2.89 ± 0.38) and also in primates (3.15 ± 0.73; Wilcoxon, *p* < 0.02 for all comparisons). N_CB_/N_CX_ ratios do not vary significantly across species with numbers of neurons in the rest of brain in any clade (Spearman correlation, all *p* > 0.2), which supports the conclusion that the cerebellum and the cerebral cortex gain neurons coordinately, in a linear fashion, in all clades examined.

However, the finding that artiodactyls and glires share a similar range of N_CB_/N_CX_ ratios, which is significantly higher than the N_CB_/N_CX_ ratios in primates and afrotherians, suggests that clades differ in the precise ratio between neurons in the two structures. In this case, the lack of a shared pattern between at least afrotherians and glires precludes the inference of the ancestral scaling rules. Instead, we suggest that each clade may have its own particular N_CB_/N_CX_ ratio, which is maintained as these structures gain neurons. The evolution of the N_CB_/N_CX_ ratio thus appears to have been both concerted (in the proportional scaling of numbers of neurons in the two structures in all clades) and in mosaic (in the exact ratio within each clade, which is however maintained as numbers of neurons vary across species).

Most importantly, however, the finding of N_CB_/N_CX_ ratios that do not decrease as the rest of brain grains neurons indicates that the relative expansion in numbers of neurons in the cerebral cortex over the rest of brain in primates and artiodactyls is matched by a similar expansion of the neuronal population in the cerebellum. This concerted addition of neurons to the cerebral cortex and cerebellum is consistent with the findings that, in primates, the cerebellum, neocortex, vestibular nuclei and relays between them exhibit concerted volumetric evolution, even after removing the effects of change in other structures (Whiting and Barton, 2003), and that increases in the volume of the prefrontal cerebral cortex are accompanied by increases in the volume of the prefrontal cortico-pontine system and prefrontal-projecting cerebellar lobules (Ramnani et al., 2006; Balsters et al., 2010). The concerted scaling of numbers of neurons in the cerebral cortex and cerebellum also agree with recent models of brain function that consider that these two structures work in conjunction (Leiner et al., 1989; Ramnani, 2006; Ito, 2008), instead of endorsing a functional preponderance of the cerebral cortex over the cerebellum.

**Figure 12:**
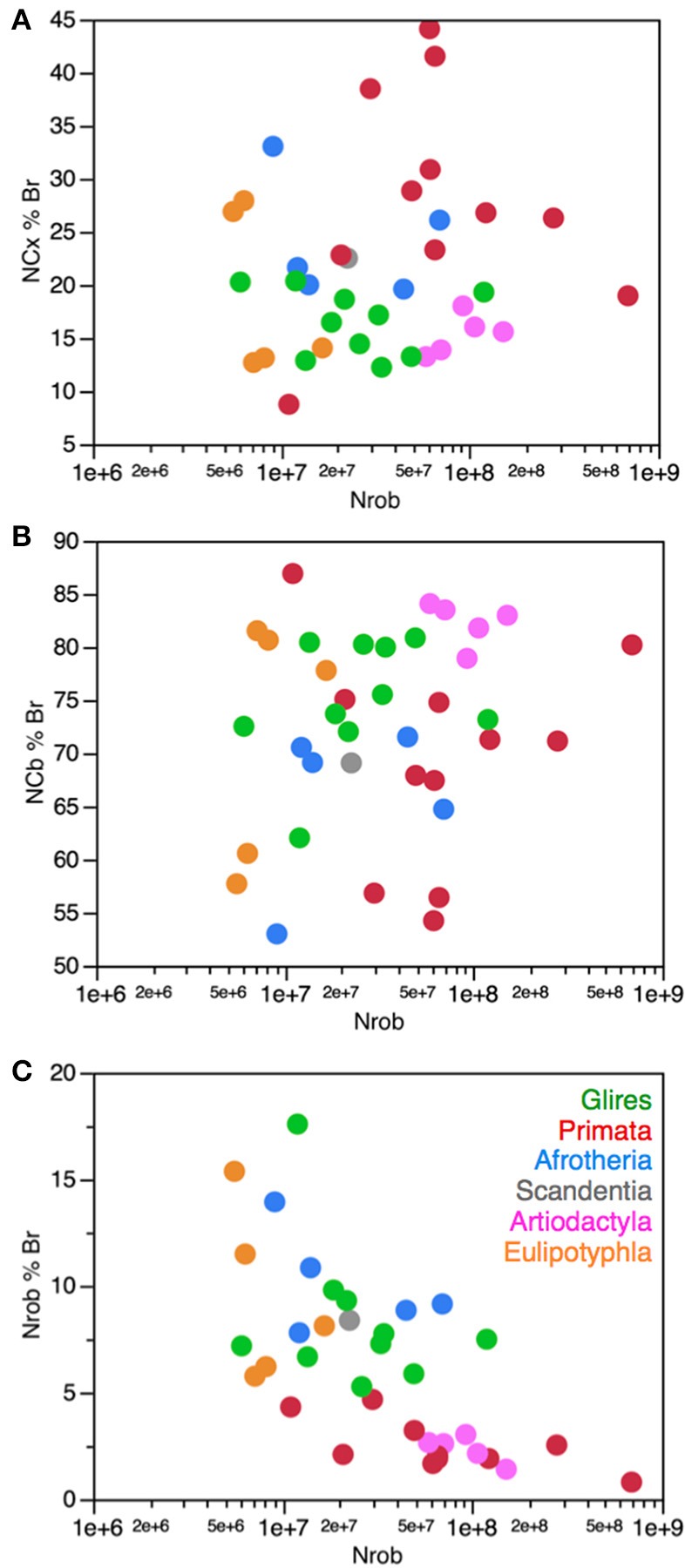
**No systematic variation in relative number of brain neurons in the cerebral cortex and cerebellum with variations in the number of neurons in the rest of brain**. Each symbol represents the average values for the structures indicated in one species (afrotherians, blue; glires, green; eulipotyphlans, orange; primates, red; scandentia, gray; artiodactyls, pink). **(A)** The percentage of brain neurons found in the cerebral cortex in each species does not vary in correlation with the number of neurons in the rest of brain (Spearman correlation, *p* = 0.5242). **(B)** The percentage of brain neurons found in the cerebellum in each species also does not vary in correlation with the number of neurons in the rest of brain (Spearman correlation, *p* = 0.3838). **(C)** The percentage of neurons in the rest of brain, however, decreases significantly with increasing number of neurons in the rest of brain (Spearman correlation, ρ = −0.665, *p* < 0.0001).

The coordinate addition of neurons to the cerebral cortex and cerebellum in evolution is further confirmed by the finding that while the relative number of brain neurons located in the rest of brain decreases significantly with increasing absolute numbers of neurons in the rest of brain (as the cerebral cortex and cerebellum gain disproportionately more neurons than the rest of brain; Figure 12C), the relative number of brain neurons found separately in the cerebral cortex and in the cerebellum does not vary significantly (Figures 12A,B). Thus, brain evolution in primates and artiodactyls can no longer be equated simply with the relative neuronal expansion of the cerebral cortex, but rather with the faster expansion of numbers of neurons in both the cerebral cortex and cerebellum relative to the rest of brain, concertedly across all species and clades, even if there is some level of mosaicism in the exact N_CB_/N_CX_ ratio observed in each clade.

### Relative allocation of numbers of neurons to the olfactory bulb and rest of brain

While the cerebral cortex and the cerebellum always have more neurons than the rest of brain in the species in our sample, the ratio between numbers of neurons in the olfactory bulb and in the rest of brain, N_OB_/N_ROB_, is typically smaller than 1, indicating fewer neurons in the olfactory bulb than in the rest of brain (Figure 10C). It is only in eulipotyphlans that the N_OB_/N_ROB_ is larger than 1, with an average value of 1.52 ± 0.31. This is significantly larger than the average N_OB_/N_ROB_ in glires (0.65 ± 0.11), which in turn is significantly larger than the average N_OB_/N_ROB_ in artiodactyls (0.23 ± 0.05; Wilcoxon, *p* = 0.0329 and *p* = 0.0253, respectively; Figure 10C). The average N_OB_/N_ROB_ in primates (0.49 ± 0.27) is not significantly different from that found in any other clade (Wilcoxon, all *p* > 0.1; Figure 10C). Humans, whose olfactory bulb we have estimated to contain 15–16 million neurons (Ribeiro et al., 2014), presumably have an N_OB_/N_ROB_ ratio of 0.02, smaller than all other values in our dataset, in contrast to ratios of 2.09 and 2.35 in the eastern mole and hairy-tailed mole, respectively, the largest in our dataset (Figure 10C). The N_OB_/N_ROB_ ratio is not significantly correlated with the number of neurons in the rest of brain in any clade (Spearman, *p* > 0.1).

The finding that artiodactyls, glires, and eulipotyphlans have significantly different N_OB_/N_ROB_ ratios, while these ratios are overlapping among glires, afrotherians, and scandentia suggests that numbers of neurons were added in similar rates to the olfactory bulb and rest of brain in the ancestral mammals, at ratios that ranged similarly to modern glires and afrotherians. Eulipotyphlans may have diverged with an increase allocation of neurons to the olfactory bulb relative to the rest of brain, while artiodactyls and primates may have diverged, to the contrary, with a decrease in the allocation of neurons to the olfactory bulb relative to the rest of brain, simultaneous to an uncoupling of numbers of neurons allocated to either structure.

Curiously, all of these mammalian clades have overlapping numbers of neurons in the olfactory bulb, varying between 2.1 and 58.1 million, as can be seen in Figure 8C. The overlap across clades within a 28-fold range in numbers of neurons in the face of much wider ranges of numbers of neurons in the rest of brain (123-fold), cerebral cortex (1649-fold) and cerebellum (3301-fold) suggests that there is an evolutionary limitation to the number of neurons that can compose an olfactory bulb. Because the mass of the olfactory bulb varies much more, by 693-fold (between 0.008 g in the marmoset and 5.546 g in the greater kudu), the limitation, if any, would apply to numbers of neurons only, and not to the size of the structure. Thus, it is unlikely that connectivity and conduction time are factors in this case. Rather, one possibility is that the proliferative potential of the population of progenitor cells that give rise to the olfactory bulb in development and throughout life is limited, thus curtailing the final, adult number of neurons that can compose the olfactory bulb. Such a limitation would also explain the uncoupling of the numbers of neurons allocated to the olfactory bulb from the numbers of neurons allocated to the rest of brain, which are largest in primates and artiodactyls (Figure 11).

### Relative allocation of numbers of neurons to the olfactory bulb and cerebral cortex

It was once suggested that the cerebral cortex expansion in evolution occurred at the expense of the olfactory bulb (Stephan and Andy, 1969). In contrast, and as mentioned above, we have recently reported that the olfactory bulb gains neurons at a greater rate than the cerebral cortex in eulipotyphlans (Figure 9A; Ribeiro et al., 2014). Across clades, the ratio between numbers of neurons in the olfactory bulb and in the cerebral cortex, N_OB_/N_CX_, is typically smaller than 1, indicating fewer neurons in the olfactory bulb than in the cerebral cortex (Figure 11B). This ratio is largest in eulipotyphlans, with an average value for the clade of 0.75 ± 0.16 that is significantly larger than the average N_OB_/N_CX_ in glires (0.30 ± 0.04), which in turn is significantly larger than the average N_OB_/N_CX_ both in artiodactyls (0.03 ± 0.01) and in primates (0.10 ± 0.06; Wilcoxon, *p* = 0.0234, *p* = 0.0200, and *p* = 0.0216, respectively; Figure 11B). The average N_OB_/N_CX_ in primates is also significantly smaller than in glires (*p* = 0.0329), but is not significantly different from artiodactyls (*p* = 0.9025).

As found for the N_OB_/N_ROB_ ratio, it is again only in eulipotyphlan species that the N_OB_/N_CX_ reaches values larger than 1, with ratios of 1.07 and 1.21 in the hairy-tailed mole and in the eastern mole, respectively, the largest in our dataset (Figure 11B). This means that these species have more neurons in the olfactory bulb than they do in the entire cerebral cortex: 16.8 million in the olfactory bulb compared to 15.7 million neurons in the cerebral cortex of the hairy-tailed mole, and 34.6 million vs. 28.7 million neurons in the eastern mole. This is in stark contrast to the largest primates: although in the mouse lemur we find 7.6 million neurons in the olfactory bulb compared to 22.3 million neurons in the cerebral cortex, with an N_OB_/N_CX_ ratio of 0.34, in humans we estimate 15–16 million neurons in the olfactory bulb (Ribeiro et al., 2014) compared to an average of 16 billion neurons in the cerebral cortex, with a putative N_OB_/N_CX_ ratio of 0.001, smaller than all other values in our dataset.

In contrast to the N_OB_/N_ROB_ ratio, which is not significantly correlated with the number of neurons in the rest of brain in any clade (Spearman, *p* > 0.1), the N_OB_/N_CX_ ratio increases significantly in concert with increased numbers of neurons in the rest of brain across eulipotyphlans (Spearman correlation, ρ = 0.900, *p* = 0.0374), but it decreases with increasing numbers of neurons in the rest of the brain across primate species (Spearman ρ = −0.900, *p* = 0.0374). The power functions relating N_OB_/N_CX_ and numbers of neurons in the rest of brain do not, however, reach significance within these clades (*p* = 0.1374 and 0.0500, respectively).

The finding that artiodactyls and primates, glires and eulipotyphlans have significantly different N_OB_/N_CX_ ratios, while these ratios are overlapping among glires, afrotherians and scandentia, suggests that numbers of neurons were added in similar rates to the olfactory bulb and cerebral cortex in the ancestral mammals, at ratios that ranged similarly to those found in modern glires and afrotherians. Eulipotyphlans may have diverged with an increase allocation of neurons to the olfactory bulb relative to the cerebral cortex (as well as relative to the rest of brain), while artiodactyls and primates may have diverged, to the contrary, with a decrease in the allocation of neurons to the olfactory bulb relative to the cerebral cortex (and rest of brain). In the evolution of the olfactory bulb, we therefore also find evidence of both concerted and mosaic scaling with the cerebral cortex in regard to the allocation of numbers of neurons to either structure.

## The consequence of scaling of numbers of neurons and average neuronal cell size: scaling of mass across brain structures

**Figure 13:**
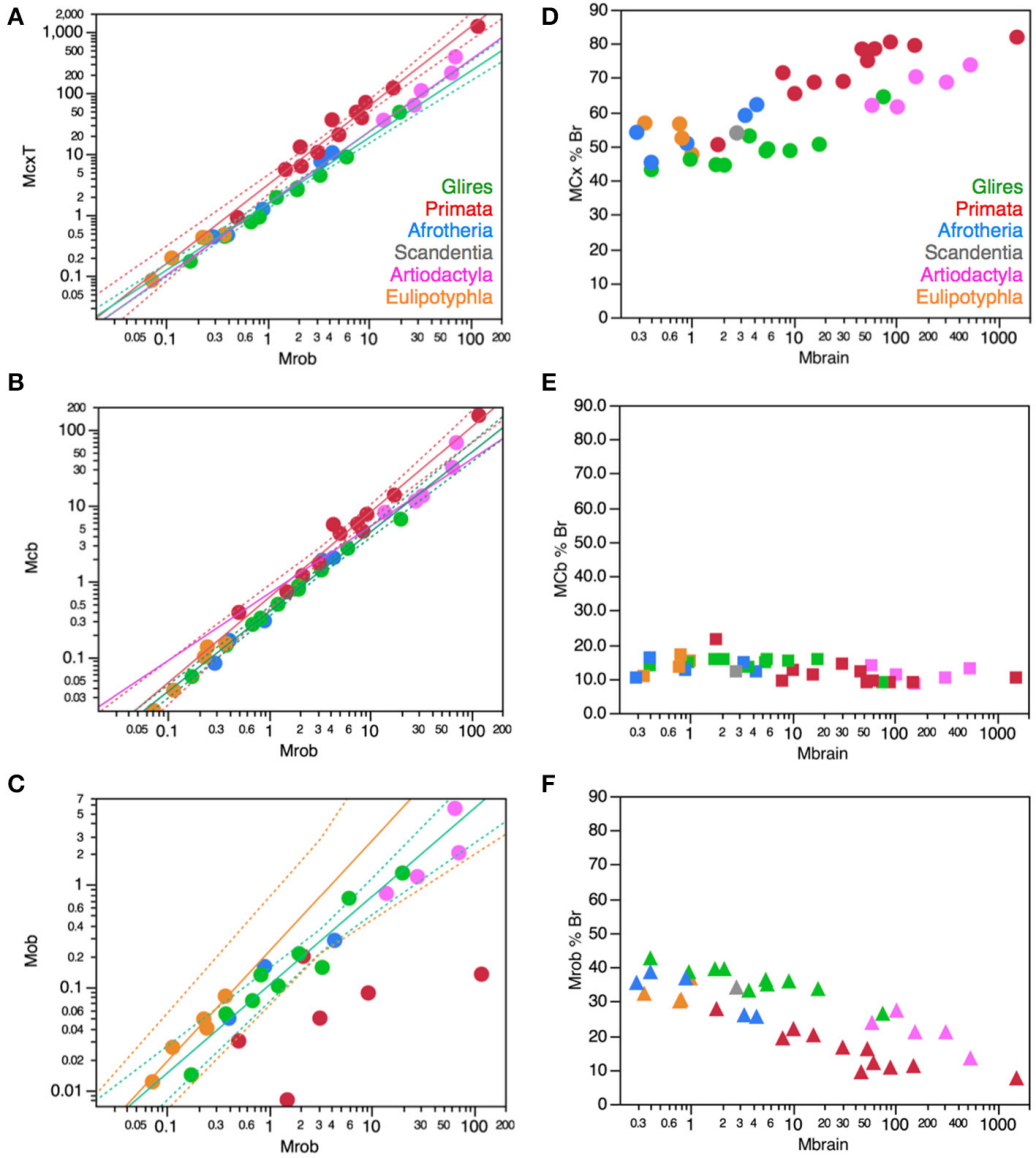
**Absolute and relative mass relationships across brain structures**. Each symbol represents the average values for the structures indicated in one species (afrotherians, blue; glires, green; eulipotyphlans, orange; primates, red; scandentia, gray; artiodactyls, pink). **(A)** the mass of the cerebral cortex increases more rapidly with increasing rest of brain mass across primates (exponent, 1.294 ± 0.069, *p* < 0.0001; red) than across all other clades (exponent, 1.136 ± 0.025, *p* < 0.0001; green). **(B)** The mass of the cerebellum also increases more rapidly with increasing rest of brain mass across primates (exponent, 1.123 ± 0.067, *p* < 0.0001; red) than across all other clades (exponent, 1.046 ± 0.019, *p* < 0.0001; green). **(C)** the mass of the olfactory bulb increases similarly across non-primate clades with increasing rest of brain mass (exponent, 0.808 ± 0.041, *p* < 0.0001; green), in a relationship that excludes primates, but the exponent for eulipotyphlans is significantly higher (1.076 ± 0.137, *p* = 0.0043; orange). **(D)** The percentage of brain mass found in the cerebral cortex varies across all species in correlation with total brain mass (Spearman correlation, ρ = 0.7551, *p* < 0.0001), and primates have a relatively larger cerebral cortex than other mammals of similar brain mass. **(E)** The percentage of brain mass found in the cerebellum varies across all species in negative correlation with total brain mass (Spearman correlation, ρ = −0.4948, *p* = 0.0019). **(F)** The percentage of brain mass found in the rest of brain decreases with increasing brain mass across all species (Spearman correlation, ρ = −0.7807, *p* < 0.0001), and is smaller in primates than in glires and artiodactyls of similar brain mass.

Comparative studies traditionally examine the absolute or relative mass of the cerebral cortex and cerebellum as functions of total brain mass (e.g., Finlay and Darlington, 1995; Barton and Harvey, 2000; Clark et al., 2001). These relationships are shown for our dataset in Figure 13, depicting the variation in the absolute mass of each brain structure as a function of the mass of the rest of brain (Figures 13A–C), and the relative mass of each brain structure as a function of total brain mass (Figures 13D–F). Now that data on numbers of neurons in these structures are available, it can be appreciated how mass relationships are confounded by different neuronal scaling rules across structures and clades.

Judging solely from the scaling of cerebral cortical mass with the mass of the rest of brain, it would appear that it is only in primates that there is a faster scaling of the cerebral cortex relative to the rest of brain (Figure 13A). In primates, this relationship has an exponent of 1.294 ± 0.069 (*p* < 0.0001; Figure 13A, red), which excludes the artiodactyl datapoints, and is significantly larger than the exponent that applies to all other clades, including artiodactyls (1.136 ± 0.025, *p* < 0.0001). However, as shown in Figure 8A, the number of neurons in the artiodactyl cerebral cortex also scales faster relative to the number of neurons in the rest of brain compared to other non-primate species. The discrepancy, in this case, is due to the differential scaling of the mass of the rest of brain as the structure gains neurons in artiodactyls compared to all other clades (Figure 5). Mass relationships are thus confounded by the different neuronal scaling rules that apply to different brain structures and clades, and cannot be used as proxies for the scaling of neurons across structures.

A similar scenario is found for the scaling of cerebellar mass with rest of brain mass, which again differs only between primates and non-primates (exponents, 1.123 ± 0.067 and 1.046 ± 0.019, both *p* < 0.0001; Figure 13B), while direct analysis of numbers of neurons in the structures shows that artiodactyls also exhibit a faster increase in numbers of neurons in the cerebellum relative to the rest of brain (Figure 5B). Also in the olfactory bulb, structure mass might appear to scale similarly with rest of brain mass across non-primate clades (with a joint exponent of 0.808 ± 0.041, *p* < 0.0001; Figure 13C), masking the much faster increase in numbers of olfactory bulb neurons over rest or brain neurons in eulipotyphlans compared to other clades (Figure 8C).

Consistently with the faster increase in cortical mass over rest of brain mass shown above, and as found before (Frahm et al., 1982; Finlay and Darlington, 1995; Clark et al., 2001), there is a significant positive correlation across species in all clades between total brain mass and the relative mass of the cerebral cortex, indicative of a relative expansion in cortical mass (Spearman correlation, ρ = 0.7551, *p* < 0.0001; Figure 13D), which occurs at the expense of the relative mass of the rest of brain, whose relative mass decreases with increasing brain mass across all species (Spearman correlation, ρ = −0.7807, *p* < 0.0001; Figure 13F). However, contrary to reports that the relative mass of the cerebellum is stable across species (Clark et al., 2001), we also find a significant negative correlation between the relative mass of the cerebellum and increasing brain mass across all species (Spearman correlation, ρ = −0.4948, *p* = 0.0019; Figure 13E). This type of analysis reinforces the notion of a relative expansion of the cerebral cortex in mammalian brain evolution that is particularly pronounced in primates: within each clade, it is only in primates and glires that larger brains have significantly relatively larger cerebral cortices (Spearman correlation, ρ = 0.9273 and 0.8182, *p* < 0.0001 and *p* = 0.0038, respectively; other clades, including artiodactyls, *p* > 0.1), and for comparable brain masses, the cerebral cortex is relative larger in primates than in glires (Figure 13D, red and green).

Although using brain mass as an independent variable has great descriptive value, it wrongly implies that total brain mass also is determinant of the mass of its parts, when mechanistically it is necessarily the other way around. To gain insight into the changes that shaped brain evolution, it is more useful to acknowledge that brain mass is the result of the sum of the masses of its parts—and each of these, in turn, results from the product between their numbers of cells and the average mass of these cells, both neurons and non-neuronal cells. Because the mass of all brain structures varies as a similar function of non-neuronal cells, as shown in Figure 2, variations in brain structure mass can be described mathematically simply as functions of their numbers of neurons compounded by variations in the average mass of these neurons. Now, because average neuronal cell mass can be inferred from neuronal density raised to the power of −1.004 (Mota and Herculano-Houzel, under review), and neuronal density in turn varies as different but known functions of numbers of neurons across structures and clades, such that neuronal density is proportional to N^d^, then the mass of brain structures, M_STR_, can be described to vary with N^1^_STR_ × N^−1.004d^_STR_, that is, N^1−1.004d^_STR_.

**Figure 14:**
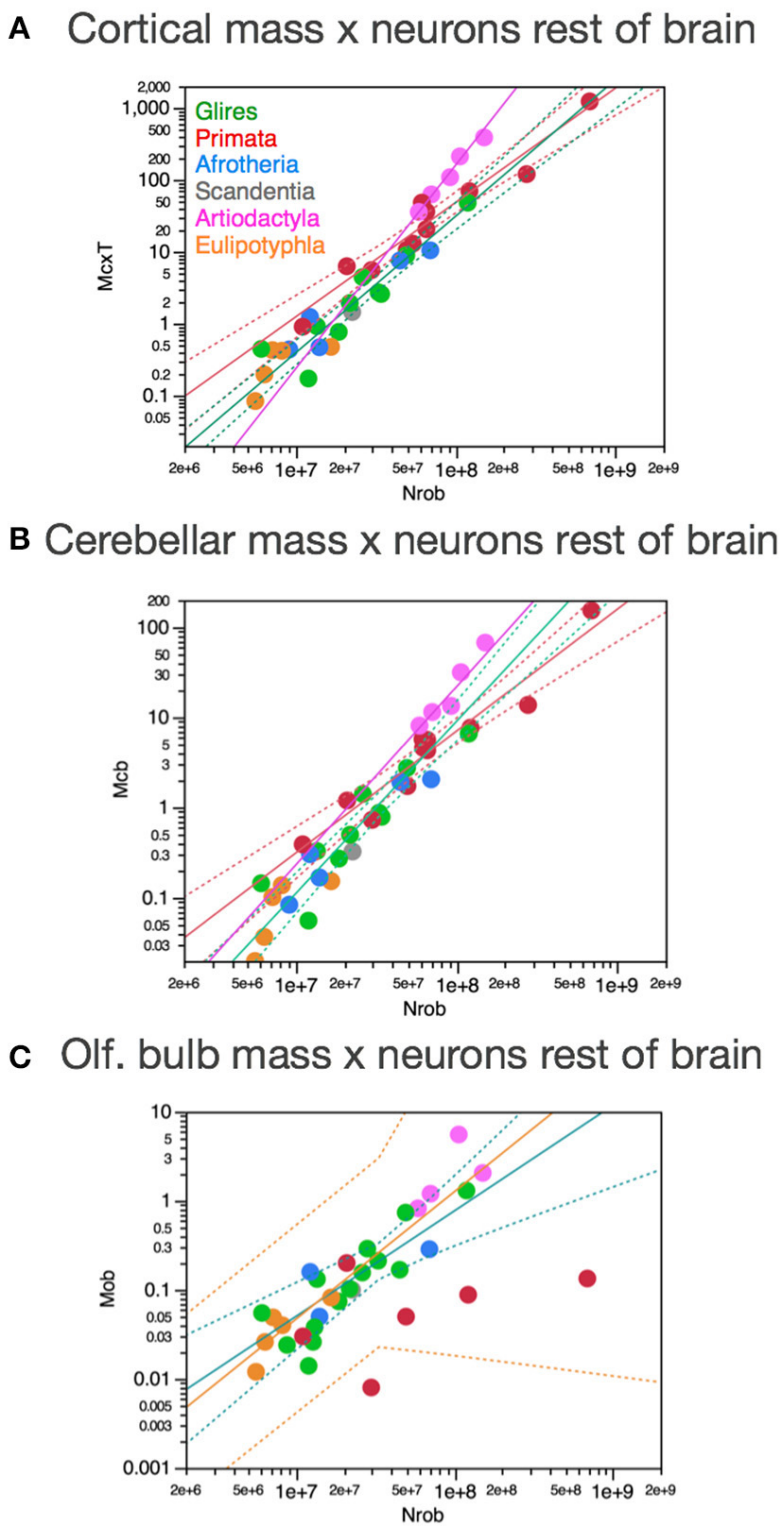
**Scaling of brain structure mass as a function of numbers of neurons in the rest of brain**. Each symbol represents the average values for the structures indicated in one species (afrotherians, blue; glires, green; eulipotyphlans, orange; primates, red; scandentia, gray; artiodactyls, pink). **(A)** The cerebral cortex gains mass much faster in artiodactyls than in other clades as a function of numbers of neurons in the rest of brain across species. Power functions plotted have exponents 2.826 ± 0.302, *p* < 0.0001 (artiodactyls, in pink), 1.588 ± 0.134, *p* < 0.0001 (primates, in red), and 1.743 ± 0.147, *p* = 0.0112 (afrotherians, glires, scandentia and eulipotyphlans, in green). **(B)** The artiodactyl cerebellum also gains mass much faster than the cerebellum in other clades as a function of numbers of neurons in the rest of brain across species. Power functions plotted have exponents 1.976 ± 0.648, *p* = 0.0929 (artiodactyls, in pink), 1.351 ± 0.126, *p* < 0.0001 (primates, in red), and 1.665 ± 0.147, *p* < 0.0001 (afrotherians, glires, scandentia and eulipotyphlans, in green). **(C)** Scaling of olfactory bulb mass as a function of numbers of neurons in the rest of brain with similar exponents of 1.449 ± 0.524 in eulipotyphlans (*p* = 0.0720) and 1.183 ± 0.249 (*p* = 0.0006) in afrotherians, glires and scandentia, while primates fall outside the 95% confidence interval for the latter.

Moreover, given the tight correlations found between numbers of neurons across different brain structures and the rest of brain, it also is possible to describe variations in the mass of each brain structure as a function of numbers of neurons in the rest of brain (Figure 14). This analysis of our dataset shows for instance that the cerebral cortex of artiodactyls gains mass as a function of the number of neurons in the rest of brain that is much steeper than in primates, falling well outside the 95% confidence interval (exponents, 2.826 ± 0.302 in artiodactyls, 1.588 ± 0.134 in primates, 1.743 ± 0.147 in all other clades, *p* < 0.0001, *p* < 0.0001, and *p* = 0.0112, respectively; Figure 14A, pink). Similarly, cerebellar mass increases more steeply in artiodactyls (exponent, 1.976 ± 0.648, although *p* = 0.0929 because of the small n) than in afrotherians, glires, eulipotyphlans and scandentia (exponent, 1.665 ± 0.147, *p* < 0.0001), and more rapidly in these than in primates (exponent, 1.351 ± 0.126, *p* < 0.0001), resulting in a much larger cerebellar mass in artiodactyls than in other clades for similar numbers of neurons in the rest of the brain (Figure 14B; notice that, despite the large *p*-value for artiodactyls, all data points fall outside the 95% confidence interval for other clades). In contrast, there is no significant difference in how the mass of the olfactory bulb increases as the rest of brain gains neurons across eulipotyphlans and the ensemble of afrotherians, glires and scandentia (exponents, respectively 1.429 ± 0.524, *p* = 0.0720 and 1.183 ± 0.249, *p* = 0.0006), although several primate species fall below the 95% confidence interval for afrotherians, glires and scandentia (Figure 14C).

**Figure 15:**
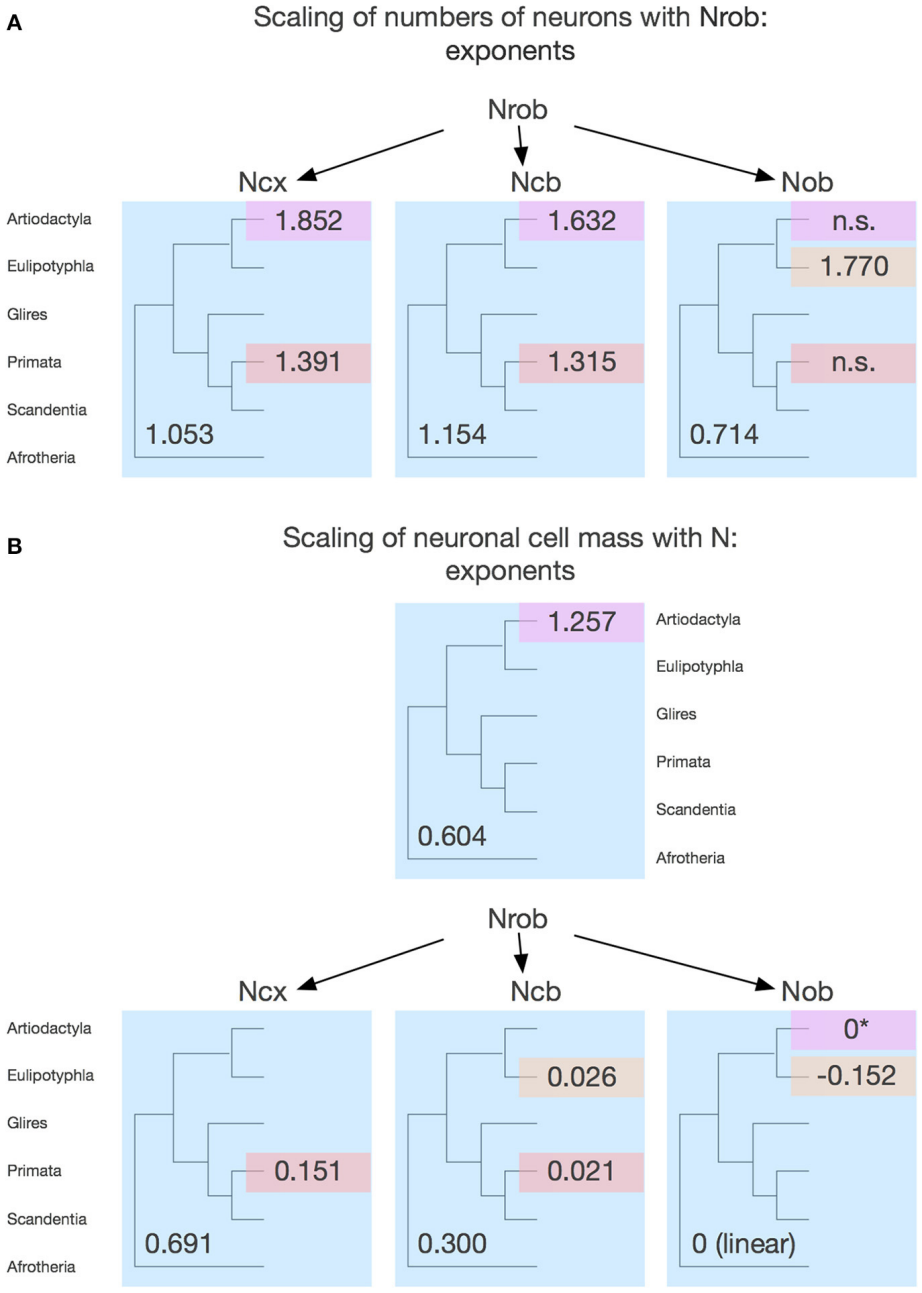
**Schematic of the proposed conserved and mosaic evolution of mammalian brain scaling. (A)** Scaling of numbers of neurons in the different brain structures as the rest of brain gains neurons. The three panels show the phylogenetic trees indicating in blue what we propose to be the scaling exponents that applied to ancestral mammals determining the rate at which each structure gains neurons as the rest of brain also gains neurons, and that still apply to some modern clades. The different colors show the clade-specific changes in the respective exponents. n.s., exponent is non-significant. Notice that both artiodactyls and primates exhibit a change in rate over the ancestral scaling that is however coordinated across cerebral cortex and cerebellum. **(B)** Scaling of estimated average neuronal cell mass in the different brain structures as each structure gains neurons. The four panels show the phylogenetic trees indicating in blue what we propose to be the scaling exponents that applied to ancestral mammals determining the rate at which neurons in each structure increase in size (mass) as the structure gains neurons, that still apply to some modern clades, and in different colors the exponents that apply to divergent clades. The asterisk for the artiodactyl olfactory bulb indicates that although there is still no significant scaling in the structure, as in the putative ancestral scaling rules, average neuronal cell mass is inferred to have undergone a step increase relative to the ancestral condition.

The actual scaling relationships between brain structure mass and numbers of neurons in the rest of brain can be predicted from the combination of (1) scaling of numbers of neurons in each brain structure as a function of numbers of neurons in the rest of brain and (2) scaling of average neuronal cell mass in each structure as a function of its number of neurons, estimated from the scaling of neuronal cell densities as described above. Figure 15A schematizes how numbers of neurons in the cerebral cortex, cerebellum and olfactory bulb are found to scale as functions of the number of neurons in the rest of brain in what we propose that was the ancestral scaling rules (maintained in modern glires, afrotherians, and additional clades depending on the structure) and how the scaling deviated or not in the artiodactyl, primate and eulipotyphlan evolutionary branches. The coordinate changes in exponents for the cerebellum and cerebral cortex in artiodactyls and primates explain the relative expansion of numbers of neurons in these structures relative to the rest of brain while maintaining constant ratios of neurons in these structures for each clade. The scheme also illustrates the relative increase in numbers of neurons in the olfactory bulb of eulipotyphlans over both the number of neurons in the rest of brain and the cerebral cortex.

Figure 15B schematizes how average neuronal cell mass in each structure can be inferred to scale as a function of the number of neurons in the same structure in what we propose to have been the ancestral scaling rules, and how the scaling of average neuronal cell mass deviated in the primate cerebral cortex and cerebellum, in the eulipotyphlan cerebellum and olfactory bulb, and in the artiodactyl rest of brain and olfactory bulb. These exponents explain for instance how the cerebral cortex always gains mass faster than the cerebellum as these structures gain neurons coordinately; how, in both primates and artiodactyls, neurons in the rest of brain increase in mass much faster than neurons in both the cerebral cortex or cerebellum as the structures gain neurons; and how the eulipotyphlan olfactory bulb is the sole example so far of a structure in which the average mass of neuronal cells decreases as the structure gains neurons (Sarko et al., 2009).

Thus, in the light of the scaling of numbers of neurons in the cerebral cortex and cerebellum, the differential scaling of average neuronal cell mass across clades explains for instance how, within each clade, the cerebral cortex gains mass much faster than the cerebellum, but (1) despite coordinate increases in numbers of neurons in the two structures and (2) at different rates in different clades. The accelerated scaling of cortical mass over cerebellar mass in primates results from the faster increase in average neuronal cell size in the cerebral cortex than in the cerebellum as these structures gain neurons coordinately. Importantly, given the attention that the issue draws in the literature, the “relative expansion of the cerebral cortex” does not reflect an ever-increasing ratio of cortical neurons over cerebellar neurons, but it does reflect an increasing numerical preponderance of neurons in the cerebral cortex over the rest of brain—and this is the case not only in primates, but also in artiodactyls, as illustrated in Figure 10A.

As shown above, using the rest of brain as an internal reference for brain scaling can be very revealing of conserved and mosaic patterns of evolution of brain structure mass. Supplementary Figure S16 shows the predicted scaling of the mass of each brain structure as the product of (1) the scaling of numbers of neurons in each brain structure as a function of numbers of neurons in the rest of brain and (2) the scaling of average neuronal cell mass in each structure as a function of its number of neurons. We illustrate in blue how we propose, given the patterns of shared and distinctive scaling rules described throughout this manuscript, that the mass of the various brain structures scaled uniformly with numbers of neurons in the rest of brain in the mammalian ancestors prior to the divergence of primates, artiodactyls and eulipotyphlans, and we indicate in red, pink and orange how we propose that the scaling of the mass of each brain structure diverged from those ancestral rules with the branching of each of these three clades. The comparison of the predicted and measured exponents shows that both conserved and mosaic patterns of scaling of brain structure mass in mammalian evolution can be well explained by variations in the relative allocation of neurons to different brain structures, and by variations in how average neuronal cell mass scales as a function of numbers of neurons in each structure.

Our data thus indicate that the apparent primary uniformity in mass relationships across brain structures and species proposed by Finlay and Darlington (1995) does not exist. Rather, it is a consequence of changes in the scaling of numbers of neurons across structures, which may or may not be compensated by changes in the scaling of average neuronal cell size in the structures. As described in this section, this compensation is clear in the case of artiodactyls, where mass relationships mask a relative increase in numbers of cortical neurons that becomes obvious when numbers of neurons are analyzed directly.

## Scaling of brain structures with body mass

As seen with the scaling of brain structure mass with the mass of the rest of brain, the analysis of scaling of brain structure mass with body mass, shown in Supplementary Figures S17A–C, suggests that there is much more uniformity across clades than there actually is. At first glance, the mass of the rest of brain and of the cerebellum seem to be uniform functions of body mass across species and clades (Supplementary Figures S17B,C). The mass of the cerebral cortex could also be considered to vary uniformly as a function of body mass across non-primate species, while primates have larger cerebral cortices than expected for their body mass compared to other mammals (Supplementary Figure S17A). However, closer examination within each clade in separate shows that primates, artiodactyls, and the ensemble of afrotherians, glires, eulipotyphlans and scandentia have significantly different exponents relating cerebral cortical mass to body mass (cerebral cortex: primates, 0.942 ± 0.084, *p* < 0.0001; artiodactyls, 0.604 ± 0.034, *p* = 0.0031; others, 0.744 ± 0.030, *p* < 0.0001), although artiodactyl data points overlap with the distribution predicted for non-primates, non-artiodactyls. For the cerebellum, primates and other non-artiodactyls share similar exponents (0.739 ± 0.074 and 0.754 ± 0.032, respectively, both *p* < 0.0001), although with different constants, so that the distribution of cerebellar mass and body mass is not overlapping across the two groups, while artiodactyl cerebella scale with body mass raised to a smaller exponent of 0.612 ± 0.105 (*p* = 0.0252), although the distribution of artiodactyl data points also overlaps with the distribution found for non-primates, non-artiodactyls (Supplementary Figure S17B). The mass of the rest of brain varies as similar functions of body mass across primates (0.706 ± 0.076, *p* < 0.0001) and non-primate, non-artiodactyls (0.657 ± 0.022, *p* < 0.0001), but on closer inspection is found to scale differently in artiodactyls, with a smaller exponent of 0.352 ± 0.056 (*p* = 0.0242; Supplementary Figure S17C). These distinct relationships are obscured by a much looser distribution of brain structure mass as a function of body mass (Supplementary Figures S17A–C) than the distribution of brain structure mass as a function of the number of neurons in each structure (Figures 3–6). This looser distribution, with greater variation across species, supports the notion we have put forward that body mass is not a determinant of brain structure, but rather a parameter only loosely and indirectly related to the mass and numbers of neurons in the central nervous system (Burish et al., 2010).

The analysis of how numbers of neurons in the different brain structures scale with body mass clarifies the issue of the scaling of brain structure mass with body mass. This analysis shows a clear distinction indeed among primates, artiodactyls, and all other clades. The primate cerebral cortex gains neurons at a much faster rate than the ensemble of afrotherians, glires, eulipotyphlans and scandentia with increasing body mass (0.825 ± 0.097, *p* < 0.0001 against 0.402 ± 0.039, *p* < 0.0001), and artiodactyls scale with an exponent of 0.470 ± 0.087 (*p* = 0.0326) that appears to overlap with the distribution for non-primates (Supplementary Figure S17D). The same pattern applies to the scaling of cerebellum, which gains neurons at a much faster rate in primates than in non-primate, non-artiodactyls with increasing body mass (0.754 ± 0.073 vs. 0.463 ± 0.045, both *p* < 0.0001), while artiodactyls overlap with the latter (exponent, 0.452 ± 0.122, *p* = 0.0654; Supplementary Figure S17E). However, artiodactyls gain neurons in the rest of brain at a rate that is significantly lower than what applies to non-artiodactyls, non-primates (exponents, 0.234 ± 0.042 and 0.364 ± 0.039, respectively, *p* = 0.0309 and <0.0001), and primates have an even higher exponent (0.525 ± 0.089, *p* = 0.0002; Supplementary Figure S17F).

Thus, the analysis of the scaling of numbers of neurons in the artiodactyl rest of brain with increasing body mass indicates that the conformity of artiodactyls to the body-related scaling rules that apply to numbers of neurons in the cerebral cortex and cerebellum is only apparent, and not the result of a determining effect of body mass upon numbers of neurons in the two structures. Rather, according to the evolutionary scenario that we propose here, numbers of neurons in the artiodactyl cerebral cortex and cerebellum scale jointly with numbers of neurons in the rest of brain, and in a manner that differs from that found in afrotherians, glires, eulipotyphlans, and scandentia (and thus also in the common mammalian ancestors), on the one hand, and from that found in primates, on the other. The finding that artiodactyls have a different primary relationship between neurons in the rest of brain and body mass, as shown in Supplementary Figure S17F, with fewer neurons in the rest of brain than expected for their body mass according to the scaling seen in non-primates, explains how the numbers of neurons found in their cerebral cortex and cerebellum appear to conform to the rules that apply to other non-primates. In line with the notion that it is numbers of neurons in the rest of brain that should reflect any developmental relationship with the scaling of body-related functions, we thus propose that in mammalian evolution, both artiodactyls and primates diverged, in different directions, from the pattern that applies to the ensemble of modern afrotherians, glires, eulipotyphlans and scandentia, and thus presumably also applied to ancestral mammals, as illustrated in Supplementary Figure S18.

## Multivariate analysis: what determines what?

To test our proposition that scaling of brain structure mass in evolution can be explained by changes in how average neuronal cell mass relates to numbers of neurons in each structure and in how numbers of neurons are differentially allocated to each structure relative to the number of neurons in the rest of brain, with an effect of body mass only on the number of neurons in the rest of brain, if at all, we ran a principal component analysis of the four parameters in our evolutionary model of brain scaling: numbers of neurons in the structure, ratio between number of neurons in the structure and in the rest of brain, neuronal density in the structure (as a proxy for average neuronal cell mass) and body mass. In the cerebral cortex, across all 41 species we find that the factor that contributes the most to variation loads with the N_CX_/N_ROB_ ratio (0.8623) and number of neurons in the cortex (0.6601), explaining 30.1% of the variance in the data, followed by a second factor that loads with neuronal density (0.9147) and body mass (−0.4104) that explains an additional 26.1% of the variance. The same pattern applies to the cerebellum, where the factor that contributes the most to variation loads with the number of neurons in the cerebellum (0.9073) and the N_CB_/N_ROB_ ratio (0.8350), explaining 39.5% of the variance in the data, followed by a second factor that loads with neuronal density (−0.5043) and body mass (0.7923) that explains an additional 26.1% of the variance. For the olfactory bulb, the first factor, which explains 41.8% of the variance, loads with neuronal density in the structure (0.8517), followed by the N_OB_/N_ROB_ ratio (0.8250), and here also includes body mass (−0.5102). The second factor, which explains an additional 26.6% of the variance, loads with the number of neurons in the olfactory bulb (0.8754) and again the N_OB_/N_ROB_ ratio (0.4124). Body mass only loads positively and significantly in the first factor for the rest of brain, composed of body mass (0.5316), number of neurons in the structure (0.5073) and neuronal density (−0.6293), which explains 31.2% of the variance.

Multivariate analysis thus supports our proposition that the scaling of different brain structures has diverged away from the common ancestral layout through clade-specific (or clade-defining) changes in how numbers of neurons are differentially allocated to each structure relative to the number of neurons in the rest of brain, and in how average neuronal cell mass relates to numbers of neurons in each structure. Further, as proposed before (Burish et al., 2010), we posit that body mass is not a determining factor of numbers of neurons in the cerebral cortex and cerebellum, although it might have a role in determining number of neurons in the rest of brain.

## Summary: an amalgam of mosaic and concerted brain evolution

Mammalian brains vary enormously in mass and in the proportions of the structures that compose it. Here we propose, based on the direct analysis of numbers of neurons in each structure and their relationship to the mass of these structures across 41 mammalian species in 6 clades, that the diversity in mammalian brain organization in regard to the relative and absolute size (mass or volume) of its structures can be explained by clade-specific mosaic evolution in a context of otherwise concerted scaling. Moreover, we propose that the evolutionary changes that gave rise to characteristically distinct brain organization in some clades (Artiodactyla, Eulipotyphla and Primata) may have been as simple as the changes described in Supplementary Figure S19.

In the light of the current understanding of evolutionary branching of mammalian clades (Murphy et al., 2001, 2004), we interpret the similarities in the neuronal scaling rules that apply to different brain structures and modern species to infer, based on parsimony, what scaling rules applied to ancestral mammals. We propose that these animals had brains whose number of neurons in the rest of brain scaled slowly with body mass raised to an exponent of 0.364 (Supplementary Figure S18, left), and gained neurons jointly in the cerebral cortex and cerebellum as the rest of brain gained neurons maintaining N_CX_/N_ROB_ and N_CB_/_ROB_ ratios of approximately 2 and 8, with therefore approximately constant N_CB_/M_CX_ ratios of around 4. As average neuronal cell mass in these structures scaled coordinately across structures as they gain neurons, the rest of brain, cerebral cortex and cerebellum appear to scale concertedly in mass, which is simply a result of the coordinate increases in numbers of neurons and average neuronal mass in each structure. These rules would have been conserved in the lineages that gave rise to modern Glires, Scandentia, and Afrotheria (Supplementary Figure S19, blue).

In contrast, and building on these conserved rules, we identify clade-specific changes in artiodactyls, primates and eulipotyphlans that we propose are also due to evolutionary changes in the allocation of numbers of neurons to different structures and in the scaling of average neuronal mass. We propose that the differential characteristics of brain morphology in eulipotyphlans are explained by two main evolutionary changes: one that refrained neurons in the cerebellum from increases in average cell mass beyond what was present in ancestral eulipotyphlans, and another that increased the relative allocation of neurons to the olfactory bulb, changing both the N_OB_/N_ROB_ and N_OB_/N_CX_ ratios (Supplementary Figure S19, orange). All other characteristics remain as in the ancestral mammals, shared with modern mammals in other (but not all) clades. Primates, in turn, are proposed to have branched off the mammalian ancestor with step changes that increased the rate at which numbers of neurons increase with body mass (to an exponent of 0.525, from 0.364 in ancestral mammals), and caused increased N_CX_/N_ROB_ and N_CB_/_ROB_ ratios as the rest of brain gained neurons in evolution, reaching ratios of 20–27 and 40–100, respectively. A further change in the scaling of average neuronal cell mass, which stopped increasing in the cerebellum and now increased only very slowly in the cerebral cortex as these structures gained neurons, explains both how these structures come to concentrate many more neurons in primates than in other mammalian structures of similar size; and how the primate cerebral cortex expands in relative mass over the cerebellum, even though the two structures continue to gain neurons jointly (Supplementary Figure S19, red).

Importantly, we find that a relative increase in numbers of cortical (and cerebellar) neurons over the rest of brain, that is, cortical expansion, is not unique to primates. We propose that artiodactyls branched off, away from the ancestral neuronal scaling rules, with changes that slowed the scaling of numbers of neurons in the rest of brain with body size (from 0.364 in ancestral mammals to 0.234 in artiodactyls) and also increased the average size of neurons in the rest of brain. At the same time, and as in primates, another change also caused increased N_CX_/N_ROB_ and N_CB_/N_ROB_ ratios as the rest of brain gained neurons in evolution, reaching ratios of 5–11 and 26–59, respectively, with an N_CB_/N_CX_ ratio that is higher than in primates. Additionally, the branching off of artiodactyls seems to have included a relative decrease in the allocation of neurons to the olfactory bulb relative to the rest of brain (Supplementary Figure S19, pink).

While primates and artiodactyls have in common an increased ratio of neurons in the cerebral cortex and cerebellum over the rest of brain, these are not shared characteristics. First, the scaling of neurons in the cerebral cortex and cerebellum with the rest of brain differs between the two branches (Figures 3, 4, 8); and second, artiodactyls and primates have no shared evolutionary history beyond the ancestral also shared with other Euarchontoglires and Laurasiatheria clades (Murphy et al., 2001, 2004). Diversification in mammalian evolution seems to have occurred in parallel, and rapidly, with the branching of all clades analyzed here within an interval of 20 million years, between 100 and 110 million years ago (afrotherians vs. euarchontoglires and laurasitherians) and 90 million years ago (euarchontoglires vs. laurasitherians and the branching off of glires and primates, eulipotyphlans and artiodactyls within them; Murphy et al., 2004). In agreement with the current evolutionary tree of mammalian diversification, we find no evidence indicative of serial changes in brain scaling in evolution, but only of parallel evolution.

According to our model, the mechanisms of change that led to both diversity and conservation in brain scaling in eutherian evolution involved clade-specific and structure-specific modifications in the pathways that regulate cell proliferation (and presumably also cell death) that define the adult numbers of neurons, and in those pathways that regulate the volume of the soma and the extent of dendrites and axons that lead to changes in average total neuronal cell size. Although we cannot pinpoint the cellular and genetic pathways involved, our model paves the way to finding them.

## The ancestral mammalian brain

The earliest eutherian fossil known to date is *Juramaia sinensis* (Luo et al., 2011), placed 160 million years ago in the Jurassic, with an estimated body mass of 15–17 g, but unfortunately no estimate of cranial capacity. However, an earlier mammaliaform of approximately 195 million years ago is *Hadrocordium wui*, with an estimated body mass of only 2 g (Luo et al., 2001), similar to the smallest living eulipotyphlan and bat (Bloch et al., 1998). *Hadrocordium wui* is the closest known extinct relative of crown Mammalia, and had an estimated brain volume of 0.045 ml or cm^3^ (Rowe et al., 2011). Given what we propose to have been the ancestral scaling rules for mammalian brains, we can infer, from modern afrotherians and glires in the same range of brain mass, that the cerebral cortex represented ca. 45% of the brain, the rest of brain represented ca. 40%, and the cerebellum, ca. 15%, yielding approximate masses of 0.020 g, 0.018 g and 0.07 g, respectively (using the small factor relating brain mass and volume of only 1.04; Frahm et al., 1982). With these small masses, we predict that *Hadrocordium wui* had only 3.6 million neurons in the cerebral cortex, 7.5 million neurons in the cerebellum, and 2.1 million neurons in the rest of brain, which are far fewer than found in the modern species in our sample.

Insight into evolution can also be obtained in the particular case of primates from the fossil of a stem primate, *Ignacius graybullianus*, with an endocranial volume of 2.14 cm^3^ and a predicted body mass of 231 g (Silcox et al., 2009). If this species was indeed positioned close to the branching off of primates, the ancestral scaling rules for mammalian brains must still have applied. Using the assumptions above, we can infer approximate masses of 0.96, 0.86, and 0.32 g in the cerebral cortex, rest of brain and cerebellum, respectively, yielding estimates of 42.4 million, 17.2 million and 167.2 million neurons in these structures, which are in the range of those found in the modern mouse lemur (Gabi et al., 2010). Using instead the scaling rules that we propose that applied to ancestral mammals, we find fairly close values of 33.9, 18.6, and 135.3 million neurons in the cerebral cortex, rest of brain and cerebellum, respectively. The convergence between the two estimates is what we would expect in our proposed scenario of branching of primates from modifications in neuronal scaling rules and their distribution that became more and more noticeable as numbers of neurons increased across species in evolution.

## Where humans stand

We have previously reported that the human brain fits all scaling rules reported so far for primates in general: it has the expected mass for its number of neurons (Azevedo et al., 2009), the expected volume of the gray and white matter of the cerebral cortex for its number of cortical neurons (Herculano-Houzel et al., 2010), and a similar ratio between N_CB_/N_CX_ as other primate and non-primate mammals (Herculano-Houzel, 2010). Here we show that, in addition, the human brain has the ratio of numbers of neurons in the cerebral cortex to numbers of neurons in the rest of brain expected for a primate (Figure 8A), and this ratio is actually not the highest amongst primates (Figure 10A), despite a general trend toward an increase in this ratio with increasing numbers of neurons in the rest of brain in primates. Thus, we reinforce the previous conclusion that the human brain is not extraordinary within primates, given that the same scaling rules apply to it—although it is remarkable in the total number of neurons that it contains, superior to that in all other primates, possibly due to the overcoming by human ancestors of the energetic limitations that presumably curb further increases in numbers of brain neurons in non-human primates beyond what is found in extant great apes (Fonseca-Azevedo and Herculano-Houzel, 2012).

### Conflict of interest statement

The authors declare that the research was conducted in the absence of any commercial or financial relationships that could be construed as a potential conflict of interest.
