# Corrigendum: Brain scaling in mammalian evolution as a consequence of concerted and mosaic changes in numbers of neurons and average neuronal cell size

**DOI:** 10.3389/fnana.2015.00038

**Published:** 2015-03-26

**Authors:** Suzana Herculano-Houzel, Paul R. Manger, Jon H. Kaas

**Affiliations:** ^1^Instituto de Ciências Biomédicas, Universidade Federal do Rio de JaneiroRio de Janeiro, Brazil; ^2^Instituto Nacional de Neurociência Translacional, Ministério de Ciência e TecnologiaSão Paulo, Brazil; ^3^Department of Anatomy, University of the WitwatersrandJohannesburg, South Africa; ^4^Department of Psychology, Vanderbilt UniversityNashville, TN, USA

**Keywords:** brain evolution, number of neurons, cell size, number of glial cells, glia-neuron ratio

It has come to our attention that some of the data on the cellular composition of the brain of artiodactyls, presented in Table 1 of Kazu et al. (2014) and used in this review, needed minor corrections, which were published in a Corrigendum to that paper.

While those corrections do not at all modify the conclusions of the present paper, some of the power exponents reported here were influenced in minor, non-significant ways. We provide those corrected power exponents below.

p. 4, Figure 2, top right—The mass of each brain structure varies as a similar, shared power function of the number of non-neuronal (other) cells in the structure of exponent 1.050 ± 0.018 (*p* < 0.0001).

p. 6, Figure 3, top: Mass of the cerebral cortex increases with number of neurons raised to an exponent of 1.694 ± 0.048 across non-primates.

p. 6, Figure 3, bottom: Neuronal density in the cerebral cortex decreases with number of neurons raised to an exponent of −0.693 ± 0.048 (*p* < 0.0001).

p. 9, Figure 4: In non-primates, non-eulipotyphlans, cerebellar mass increases with number of neurons raised to an exponent of 1.283 ± 0.035 (*p* < 0.0001) and cerebellar neuronal density scales with number of neurons raised to an exponent of −0.282 ± 0.035 (*p* < 0.0001).

Figure 7:

A—Neuronal density in the cerebral cortex scales with neuronal density in the rest of brain raised to an exponent of 0.876 ± 0.041, *p* < 0.0001 (excludes primates).

B—Neuronal density in the cerebellum scales with neuronal density in the rest of brain raised to an exponent of 0.442 ± 0.049, *p* < 0.0001 (excludes primates and eulipotyphlans).

C—Neuronal density in the olfactory bulb scales with neuronal density in the rest of brain raised to an exponent of 0.994 ± 0.118, *p* < 0.0001 (excludes primates and eulipotyphlans).

D—Neuronal density in the olfactory bulb scales with neuronal density in the cerebral cortex raised to an exponent of 1.139 ± 0.113, *p* < 0.0001 (excludes primates and eulipotyphlans).

E—Neuronal density in the cerebellum scales with neuronal density in the cerebral cortex raised to an exponent of 0.516 ± 0.041, *p* < 0.0001 (excludes primates and eulipotyphlans).

F—Neuronal density in the olfactory bulb scales with neuronal density in the cerebellum raised to an exponent of 1.706 ± 0.161, *p* < 0.0001 (includes all clades).

p. 12, Figure 8A—Artiodactyls gain neurons in the cerebral cortex faster than they gain neurons in the rest of brain, as a power function of exponent 1.552 ± 0.056, *p* = 0.0013, *r*^2^ = 0.997 (excludes the giraffe).

p. 12, Figure 8B—Artiodactyls gain neurons in the cerebellum faster than they gain neurons in the rest of brain, as a power function of exponent 1.737 ± 0.304, *p* = 0.0107, *r*^2^ = 0.916.

p. 14, Figure 9B—The number of neurons in the cerebellum varies as a power function of the number of neurons in the cerebral cortex with an exponent of 0.922 ± 0.110, *p* = 0.0036, across artiodactyls. The relationship for the ensemble of clades can also be fit with a linear function of slope 4.16 (*p* < 0.0001, *r*^2^ = 0.985).

p. 15, Figure 10A—Artiodactyls have on average 7.35 ± 1.24 neurons in the cerebral cortex to every neuron in the rest of brain. This ratio increases as a power function of the number of neurons in the rest of brain with an exponent of 0.904 ± 0.132 (*p* = 0.0135, *r*^2^ = 0.902).

p. 16, Figure 10B—Artiodactyls have a ratio between numbers of neurons in the cerebellum and in the rest of brain of 38.32 ± 6.19.

p. 17—Artiodactyls have an average ratio of neurons in the cerebellum relative to the cerebral cortex of 5.28 ± 0.31.

p. 21, Figure 13:

A—The mass of the cerebral cortex increases across non-primates with the mass of the rest of brain raised to an exponent of 1.155 ± 0.027, *p* < 0.0001.

B—The mass of the cerebellum increases across non-primates with the mass of the rest of brain raised to an exponent of 1.054 ± 0.019, *p* < 0.0001.

C—The mass of the olfactory bulb increases across non-primates with the mass of the rest of brain raised to an exponent of 0.812 ± 0.043, *p* < 0.0001.

D—The relative mass of the cerebral cortex increases across all species in correlation with brain mass with a Spearman correlation *r*^2^ = 0.7840, *p* < 0.0001.

E—The relative mass of the cerebellum varies across all species in correlation with brain mass with a Spearman correlation *r*^2^ = −0.5270, *p* = 0.0008.

F—The relative mass of the rest of brain varies across all species in correlation with brain mass with a Spearman correlation *r*^2^ = −0.7994, *p* < 0.0001.

p. 21, Figure 14—The cerebral cortex of artiodactyls gains mass as a function of the number of neurons in the rest of brain with exponent 2.759 ± 0.145, *p* = 0.0028. The cerebellum of artiodactyls gains mass as a function of the number of neurons in the rest of brain with exponent 2.142 ± 0.492, *p* = 0.0489.

p. 24, Figure S17—In artiodactyls, cerebral cortical mass increases as a power function of body mass with exponent 0.589 ± 0.028, *p* = 0.0023; rest of brain mass increases as a power function of body mass with exponent 0.378 ± 0.056, *p* = 0.0215; the number of neurons in the cerebral cortex scales with body mass raised to an exponent of 0.454 ± 0.107, *p* = 0.0511; and the number of neurons in the rest of brain scales with body mass raised to an exponent of 0.227 ± 0.027, *p* = 0.0136.

## Conflict of interest statement

The authors declare that the research was conducted in the absence of any commercial or financial relationships that could be construed as a potential conflict of interest

