# Supplementary material for: Brain scaling in mammalian evolution as a consequence of concerted and mosaic changes in numbers of neurons and average neuronal cell size
Source: Front Neuroanat. 2014 Aug 11;8:77. doi: 10.3389/fnana.2014.00077 (PMC4127475; doi:10.3389/fnana.2014.00077)
Supplement: Supplementary file 1 [file Presentation1.ZIP › Supp Figures legends.pdf]

Figure 16. Schematic of the proposed conserved and mosaic scaling of mammalian brain structures. The four panels show the exponents describing the scaling of the mass of each brain structure as the rest of brain gains neurons, in a phylogenetic context. The trees for each brain structure indicate in blue what we propose to be the scaling exponents that applied to ancestral mammals determining the rate at which each structure gains mass as the rest of brain gains neurons, and that still apply to some modern clades. The different colors show the clade-specific changes in the respective exponents. n.s., exponent is non-significant. Exponents in large fonts were calculated by compounding the scaling of numbers of neurons in the structure as a function of numbers of neurons in the rest of brain and the scaling of estimated average neuronal cell mass in the structure as it gains neurons. Exponents in small fonts are the actual, observed exponents.

Figure 17. Scaling of brain structure mass and number of neurons with body mass. Each symbol represents the average values for the structures indicated in one species (afrotherians, blue; glires, green; eulipotyphlans, orange; primates, red; scandentia, grey; artiodactyls, pink). **a**, the mass of the cerebral cortex increases more rapidly with increasing body mass across primates (exponent, 0.942±0.084, $p<0.0001$; red) than across the ensemble of afrotherians, glires, eulipotyphlans and scandentia (exponent, 0.744±0.030, $p<0.0001$; green) and artiodactyls (exponent, 0.604±0.034, $p=0.0031$; pink). **b**, the mass of the cerebellum increases with body mass with similar exponents across primates and non-artiodactyls (exponents, 0.739±0.074 and 0.754±0.032, respectively, both $p<0.0001$), although with different constants, such that primates have more neurons in the cerebellum for a similar body mass. In contrast, the artiodactyl cerebellum scales with body mass raised to a smaller exponent of 0.612±0.105 ($p=0.0252$), although the distribution of artiodactyl data points also overlaps with the distribution found for non-primates, non-artiodactyls. **c**, the mass of the rest of brain increases similarly across primate (0.706±0.076, $p<0.0001$; red) and non-primate, non-artiodactyl clades with increasing body mass (0.657±0.022, $p<0.0001$; green), and faster than in artiodactyls (0.352±0.056, $p=0.0242$; pink). **d**, the primate cerebral cortex gains neurons with increasing body mass at a much faster rate (0.825±0.097, $p<0.0001$; red) than artiodactyls (0.470±0.087, $p=0.0326$; pink) and non-primate, non-artiodactyls (0.402±0.039, $p<0.0001$; green). **e**, the primate cerebellum also gains neurons with increasing body mass at a much faster rate (0.754±0.073, $p<0.0001$; red) than the artiodactyl (0.452±0.122, $p=0.0654$; pink) and non-primate, non-artiodactyl cerebellum (0.463±0.045, $p<0.0001$; green). **f**, artiodactyls gain neurons in the rest of brain with increasing body mass at a rate (0.234±0.042, $p=0.0309$; pink) that is significantly lower than than in non-artiodactyls, non-primates (0.364±0.039, $p<0.0001$; green) and in primates (0.525±0.089, $p=0.0002$; red).

Figure 18. Primates and artiodactyls diverged in different directions from the ancestral scaling rule relating numbers of neurons in the rest of brain and body mass. Right, scaling of number of neurons in the rest of brain as a function of body mass across species. Each symbol represents the average number of neurons in the rest of brain and body mass in one species (afrotherians, blue; glires, green; eulipotyphlans, orange; primates, red; scandentia, grey; artiodactyls, pink). We propose that the relationship shared across modern afrotherians, glires, eulipotyphlans and scandentia (green) has been conserved from the scaling rule that applied to ancestral mammals (left, areas in blue in the phylogenetic tree), and that primates and artiodactyls diverged from these ancestral rules with respectively accelerated and decelerated scaling of numbers of neurons in the rest of brain accompanying increasing body mass (arrows).

Figure 19. Summary of the proposed model of the generation of evolutionary diversity in brain scaling combining concerted and mosaic evolutionary changes in scaling rules. The areas in blue in the phylogenetic tree indicate modern clades and ancestral mammals that we propose to share the neuronal scaling rules indicated on the left. The colored areas to the right indicate the evolutionary changes in scaling rules that we propose that led to the divergence of artiodactyls, eulipotyphlans and primates from the ancestral scaling rules. cb, cerebellum; cx, cerebral cortex; rob, rest of brain; ob, olfactory bulb; Ncx, Ncb and Nrob, numbers of neurons in the cerebral cortex, cerebellum, and rest of brain, respectively; Ncx/Nrob, ratio of numbers of neurons in the cerebral cortex and in the rest of brain; Ncb/Nrob, ratio of numbers of neurons in the cerebellum and in the rest of brain; Nob/Nrob, ratio of numbers of neurons in the olfactory bulb and in the rest of brain.
