# Supplementary figures and images for: Brain scaling in mammalian evolution as a consequence of concerted and mosaic changes in numbers of neurons and average neuronal cell size
Source: Front Neuroanat. 2014 Aug 11;8:77. doi: 10.3389/fnana.2014.00077 (PMC4127475; doi:10.3389/fnana.2014.00077)

## Scaling of structure mass with Nrob: exponents

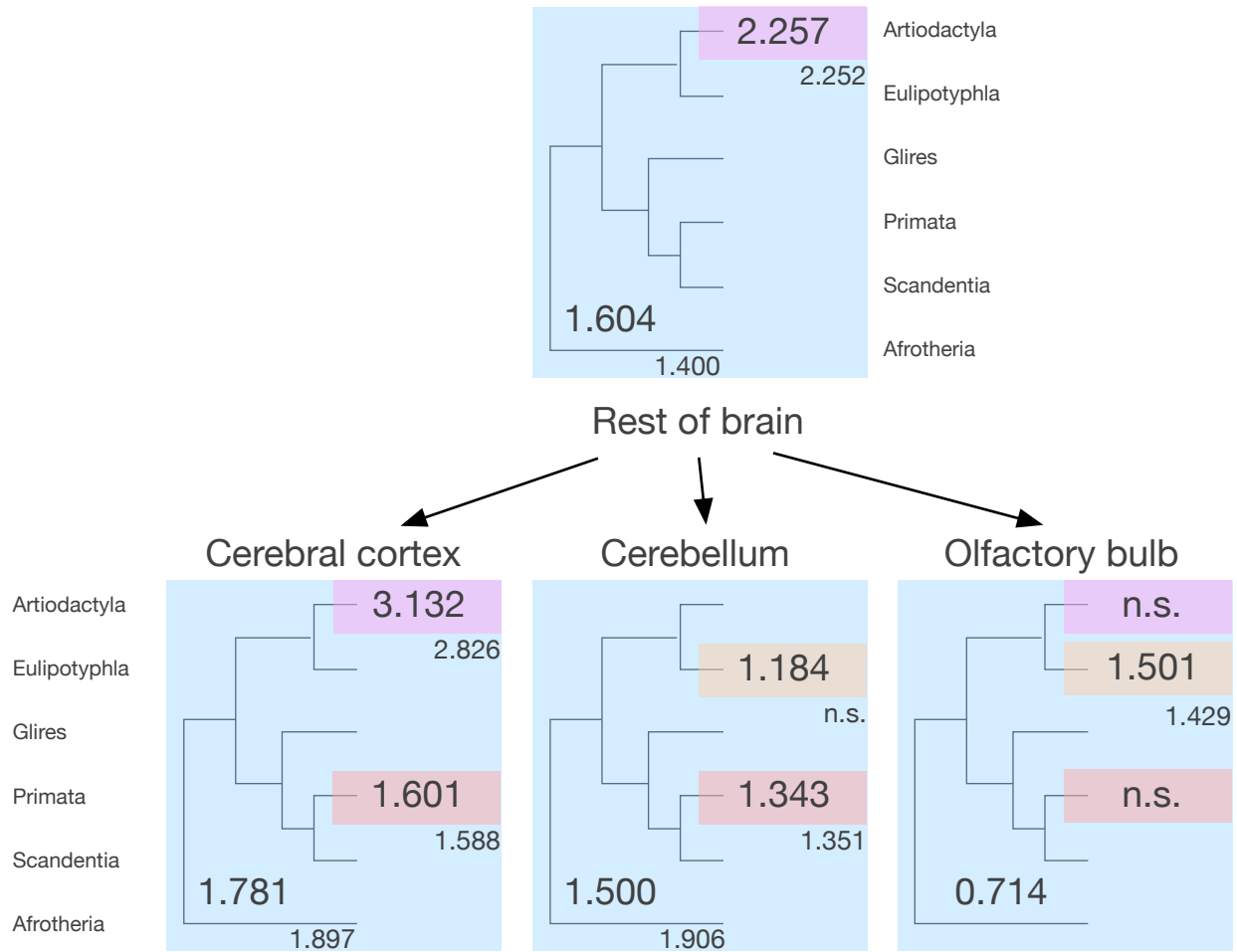

Figure 16

Supplement: Supplementary file 1 [file Presentation1.ZIP › Supp Fig 16.PDF]

a

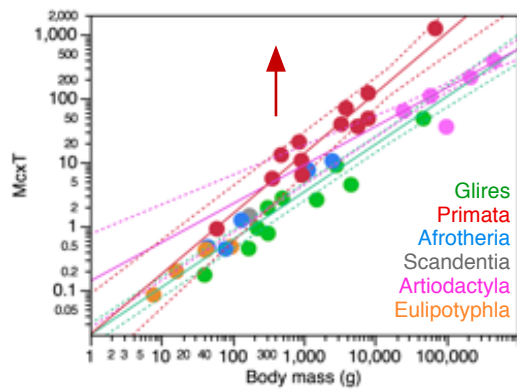

d

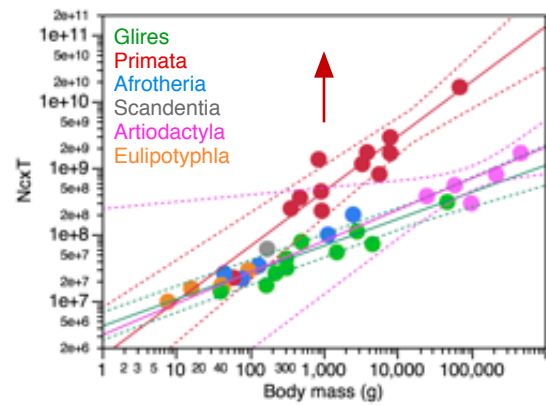

b

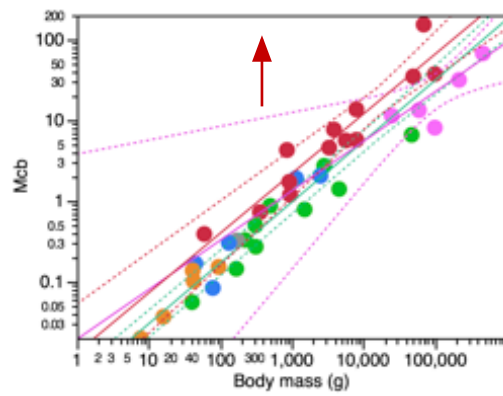

e

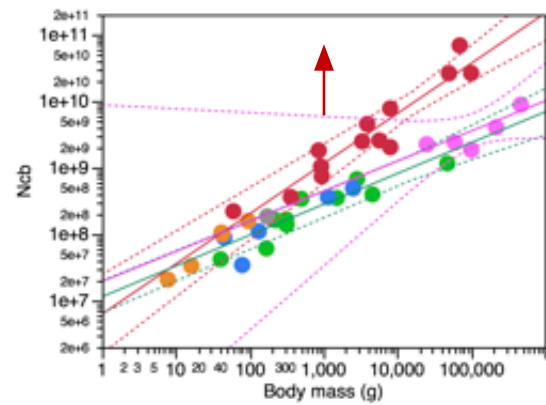

c

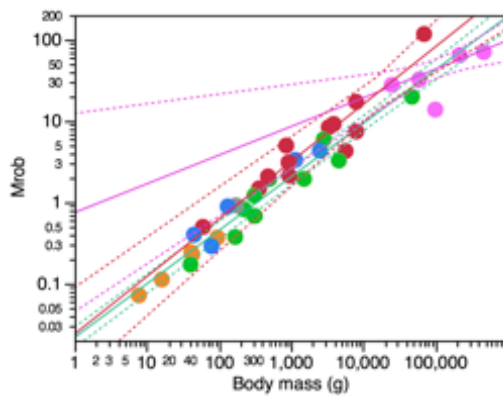

f

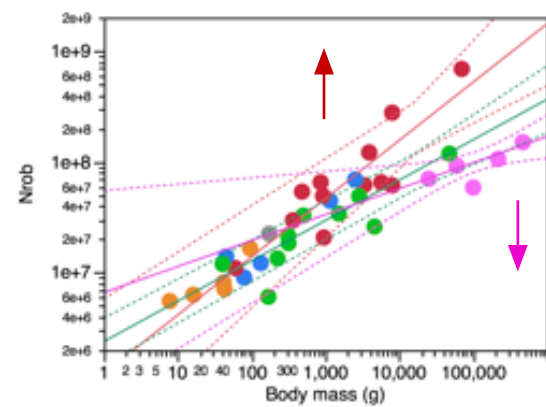

Figure 17

Supplement: Supplementary file 1 [file Presentation1.ZIP › Supp Fig 17.PDF]

Neurons in rest of brain x body mass

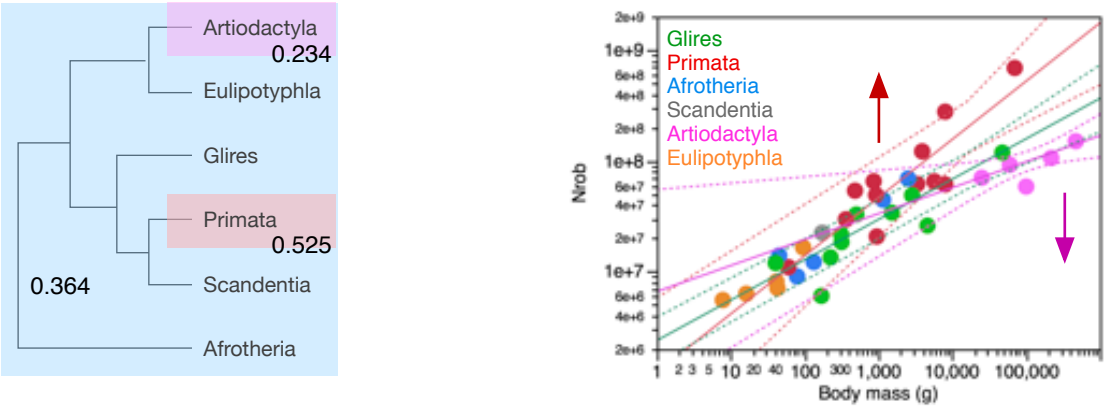

Figure 18

Supplement: Supplementary file 1 [file Presentation1.ZIP › Supp Fig 18.PDF]

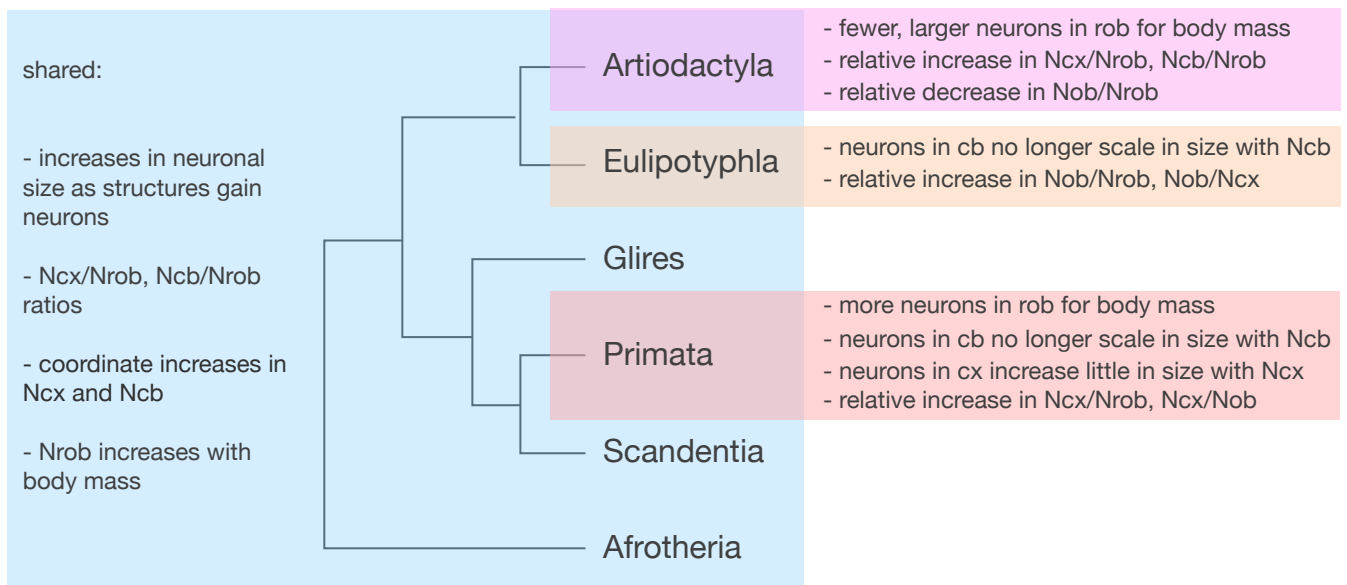

Figure 19

Supplement: Supplementary file 1 [file Presentation1.ZIP › Supp Fig 19.PDF]
